# 25-Hydroxycholesterol exacerbates vascular leak during acute lung injury

**DOI:** 10.1172/jci.insight.155448

**Published:** 2023-04-10

**Authors:** Jennifer H. Madenspacher, Eric D. Morrell, Jeffrey G. McDonald, Bonne M. Thompson, Yue Li, Konstantin G. Birukov, Anna A. Birukova, Renee D. Stapleton, Aidin Alejo, Peer W. Karmaus, Julie M. Meacham, Prashant Rai, Carmen Mikacenic, Mark M. Wurfel, Michael B. Fessler

**Affiliations:** 1 Immunity, Inflammation, and Disease Laboratory, National Institute of Environmental Health Sciences, NIH, Research Triangle Park, North Carolina, USA.; 2 Section of Pulmonary, Critical Care, and Sleep Medicine, Harborview Medical Center, Seattle, Washington, USA.; 3 Center for Human Nutrition and; 4 Department of Molecular Genetics, University of Texas Southwestern Medical Center, Dallas, Texas, USA.; 5 Department of Anesthesiology, University of Maryland School of Medicine, Baltimore, Maryland, USA.; 6 Department of Medicine, Larner College of Medicine, University of Vermont, Burlington, Vermont, USA.

**Keywords:** Inflammation, Pulmonology, Cell stress, Innate immunity, Mouse models

## Abstract

Cholesterol-25-hydroxylase (CH25H), the biosynthetic enzyme for 25-hydroxycholesterol (25HC), is most highly expressed in the lung, but its role in lung biology is poorly defined. Recently, we reported that *Ch25h* is induced in monocyte-derived macrophages recruited to the airspace during resolution of lung inflammation and that 25HC promotes liver X receptor–dependent (LXR-dependent) clearance of apoptotic neutrophils by these cells. *Ch25h* and 25HC are, however, also robustly induced by lung-resident cells during the early hours of lung inflammation, suggesting additional cellular sources and targets. Here, using *Ch25h^–/–^* mice and exogenous 25HC in lung injury models, we provide evidence that 25HC sustains proinflammatory cytokines in the airspace and augments lung injury, at least in part, by inducing LXR-independent endoplasmic reticulum stress and endothelial leak. Suggesting an autocrine effect in endothelium, inhaled LPS upregulates pulmonary endothelial *Ch25h*, and non-hematopoietic *Ch25h* deletion is sufficient to confer lung protection. In patients with acute respiratory distress syndrome, airspace 25HC and alveolar macrophage *CH25H* were associated with markers of microvascular leak, endothelial activation, endoplasmic reticulum stress, inflammation, and clinical severity. Taken together, our findings suggest that 25HC deriving from and acting on different cell types in the lung communicates distinct, temporal LXR-independent and -dependent signals to regulate inflammatory homeostasis.

## Graphical abstract

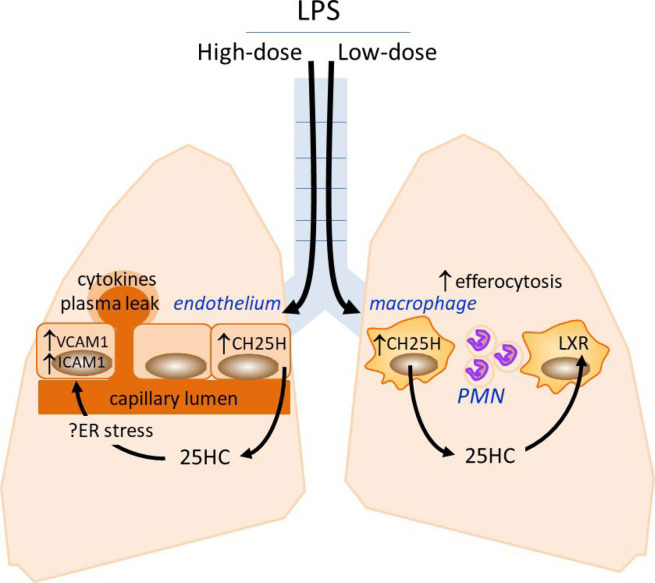

## Introduction

Oxysterols (i.e., oxidized species of cholesterol) have been studied for decades by cell biologists as feedback inhibitors of cellular cholesterol accumulation through reciprocal inhibition of the cholesterol-biosynthetic transcription factor sterol regulatory element–binding protein-2 and activation of the cholesterol-mobilizing transcription factor liver X receptor (LXR) (1). Recently, however, some oxysterols, such as 25-hydroxycholesterol (25HC), have been “rediscovered” and de-orphanized within immunology as bona fide mediators of the immune response (2). Lipopolysaccharide (LPS) dramatically induces 25HC (>80-fold) and its synthetic enzyme, cholesterol-25-hydroxylase (*Ch25h*; >100-fold), in macrophages (3–6). 25HC, in turn, feeds back to exert complex effects on the inflammatory response. It reportedly amplifies proinflammatory cytokine induction by Toll-like receptor (TLR) ligands (7), but also suppresses activation of inflammasomes (8) and NF-κB (1), and supports M2 macrophage programming (9). These reports have collectively suggested that 25HC is induced during tissue stress and may have context-dependent (tissue microenvironment–dependent) effects on inflammation and tissue homeostasis.

In mice, *Ch25h* is most highly expressed in the lung (10). In particular, *Ch25h* expression in resident alveolar macrophages (AMs) exceeds that in other tissue macrophages by more than 10-fold (11). The function of CH25H in lung biology has remained poorly defined. Recently, we reported that there is biphasic induction of *Ch25h* in distinct macrophage populations of the murine lung following low-dose LPS inhalation (2). Resident AMs upregulate *Ch25h* and 25HC within hours of LPS. By contrast, monocyte-derived AMs, which are recruited to the airspace days later during the resolution phase of inflammation, induce *Ch25h* in response to apoptotic cell encounter. We demonstrated a role for resolution-phase 25HC production by recruited AMs in LXR-dependent phagocytic clearance (“efferocytosis”) of apoptotic airspace neutrophils (PMNs) (2), but the function of the robust inflammation-phase 25HC induced by lung-resident cells has remained undefined.

Here, we used severe lung injury models (high-dose LPS, bacterial pneumonia) that better model clinical acute respiratory distress syndrome (ARDS) to probe the impact of 25HC on inflammatory lung injury. We report that, although *Ch25h^–/–^* mice exhibited a modest deficit in late-phase resolution of airspace neutrophilia, the predominant phenotype was that of marked, sustained protection from microvascular leak commencing during peak inflammation. Unexpectedly, this protection tracked with *Ch25h* deletion in non-hematopoietic cells. LPS inhalation robustly induced *Ch25h* in pulmonary endothelial cells, and CH25H-derived 25HC activated and injured the pulmonary endothelial barrier, at least in part, through endoplasmic reticulum (ER) stress that is induced in an LXR-independent manner. Suggesting relevance to humans, we show that AM *CH25H* correlates with markers of ER stress in patients with ARDS and that AM *CH25H* and bronchoalveolar lavage fluid (BALF) 25HC levels track with clinical and biochemical measures of disease severity in ARDS. Taken together, our results reveal dual roles for 25HC in the inflamed lung, with the dominant effect during acute lung injury being compromise to endothelial barrier integrity.

## Results

### Ch25h augments lung damage in severe lung injury models.

We recently reported that inhalation of low-dose LPS upregulates *Ch25h* in the lungs of mice and induces ng/mL-range 25HC in the airspace and serum of *Ch25h^+/+^* but not *Ch25h^–/–^* mice (2). Prior reports have shown that 25HC amplifies TLR ligand–induced upregulation of IL-6 and other proinflammatory cytokines via an LXR-independent mechanism (7) and is cytotoxic to endothelial cells in vitro (12). Our low-dose LPS inhalation model induces histologically mild lung inflammation. In order to better define the impact of *Ch25h* deletion on inflammatory injury in the lung, we subjected mice to a 10-fold higher concentration of LPS aerosol (3 mg/mL) (13), an exposure that more closely models acute lung injury in humans by causing much more intense and sustained inflammation (PMN peak in airway is ~5-fold higher; airway PMNs present for >120 hours rather than 48–72 hours; ref. 2).

**Figure 1:**
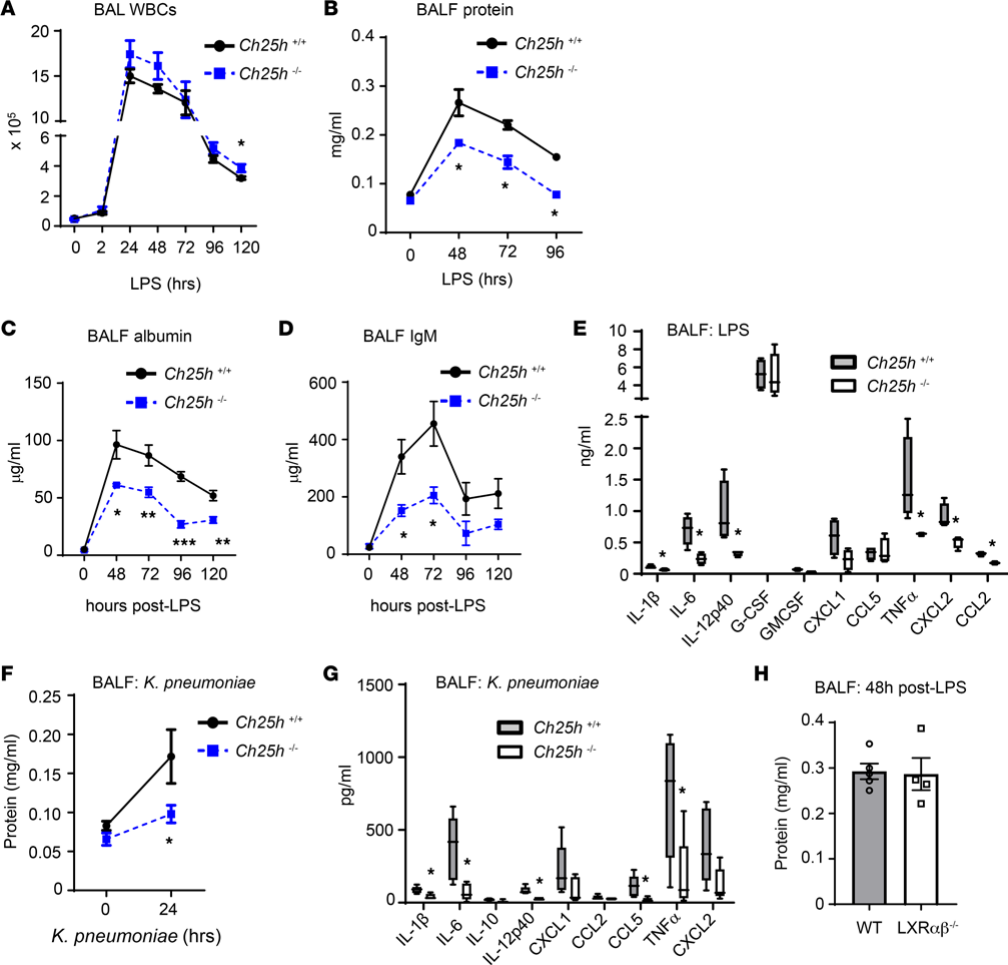
*Ch25h* deletion reduces microvascular leak and cytokines in an acute lung injury model. (**A**) BAL total leukocytes (WBCs) were quantified at various time points following a high-dose (3 mg/mL) LPS aerosol exposure (*n* = 4–9 per genotype per time point). (**B**–**D**) BALF protein (**B**), albumin (**C**), and IgM (**D**) were quantified at the indicated times following high-dose LPS aerosol (*n* = 4–6 per genotype per time point). (**E**) BALF cytokines were quantified by multiplex assay 24 hours after high-dose LPS aerosol (*n* = 5 per genotype). (**F** and **G**) Mice (*n* = 4–8 per genotype) underwent lung infection with *K*. *pneumoniae* by oropharyngeal aspiration. BALF protein (**F**) and cytokines (**G**) were quantified 24 hours after infection. (**H**) BALF protein was quantified in WT and LXR-null mice (*n* = 5 per genotype per time point, repeated twice) 48 hours after high-dose LPS inhalation. Data are mean ± SEM and are representative of 2–3 independent experiments. *P < 0.05, **P < 0.01, ***P < 0.001 by unpaired 2-tailed *t* test.

A modest deficit in late-phase clearance of alveolar neutrophilia was again observed in *Ch25h^–/–^* mice (Figure 1A and Supplemental Figure 1, A–D; supplemental material available online with this article; https://doi.org/10.1172/jci.insight.155448DS1), as in the low-dose LPS model (2). No significant differences were seen between *Ch25h^+/+^* and *Ch25h^–/–^* mice in pulmonary intravascular or extravascular (interstitial) neutrophils at 48 hours after LPS (Supplemental Figure 1, E–G), nor were there overt differences in pulmonary histopathology (Supplemental Figure 2). Notably, *Ch25h^–/–^* mice, however, exhibited a substantial and sustained reduction in BALF protein, albumin, and IgM (Figure 1, B–D), consistent with reduced pulmonary microvascular injury (14). *Ch25h^–/–^* lungs also had a reduction (*P* = 0.06) in wet-to-dry mass ratio at 24 hours after LPS, suggesting reduced pulmonary edema (Supplemental Figure 3). In addition, although *Ch25h^+/+^* and *Ch25h^–/–^* BALF had equivalent cytokine levels 2 hours after high-dose LPS (not shown), *Ch25h^–/–^* mice had a pronounced and wide-ranging reduction in BALF proinflammatory cytokines at all later time points through 72 hours after high-dose LPS challenge (Figure 1E and Supplemental Figure 4, A and B). Reduced BALF protein and cytokines were also seen in *Ch25h^–/–^* mice following *Klebsiella* *pneumoniae* (*K*. *pneumoniae*) lung infection (Figure 1, F and G), another model of severe and sustained lung inflammation (15). Neither BAL nor blood bacterial burden differed between genotypes in this setting, suggesting no overt defect of host defense in *Ch25h^–/–^* mice (Supplemental Figure 4, C and D). We previously reported that, as in the LPS inhalation model, *Ch25h^–/–^* mice have normal airspace neutrophilia at 24 hours after *K*. *pneumoniae* infection but elevated airspace neutrophilia at later time points (2). Unlike *Ch25h^–/–^* mice, LXR-null mice had BALF protein and cytokine levels equivalent to those of WT counterparts after LPS (Figure 1H and Supplemental Figure 4E). Taken together, these findings suggest that *Ch25h^–/–^* mice are relatively protected from vascular leak and cytokine induction during severe lung injury and that this occurs through an LXR-independent mechanism that appears to be dissociated from airspace neutrophilia.

### 25HC activates and injures the pulmonary vascular endothelial barrier.

Pulmonary microvascular leak can arise from cell-mediated bystander damage to alveolar capillaries from activated PMNs but can also be caused by direct effects of soluble mediators (cytokines, bioactive lipids) on the endothelium, in particular via ER stress (16, 17). 25HC itself reportedly compromises endothelial barrier function in culture (12, 18, 19) and causes ER stress via a GCN2/eIF2α/ATF4/TRIB3 pathway (17, 20, 21). Our finding of no significant differences between *Ch25h^+/+^* and *Ch25h^–/–^* lungs in inflammatory cellular infiltration after high-dose LPS (Supplemental Figures 1 and 2) suggested to us that the reduced capillary leak in *Ch25h^–/–^* lungs might stem from attenuated injury to the endothelium from soluble mediators.

**Figure 2:**
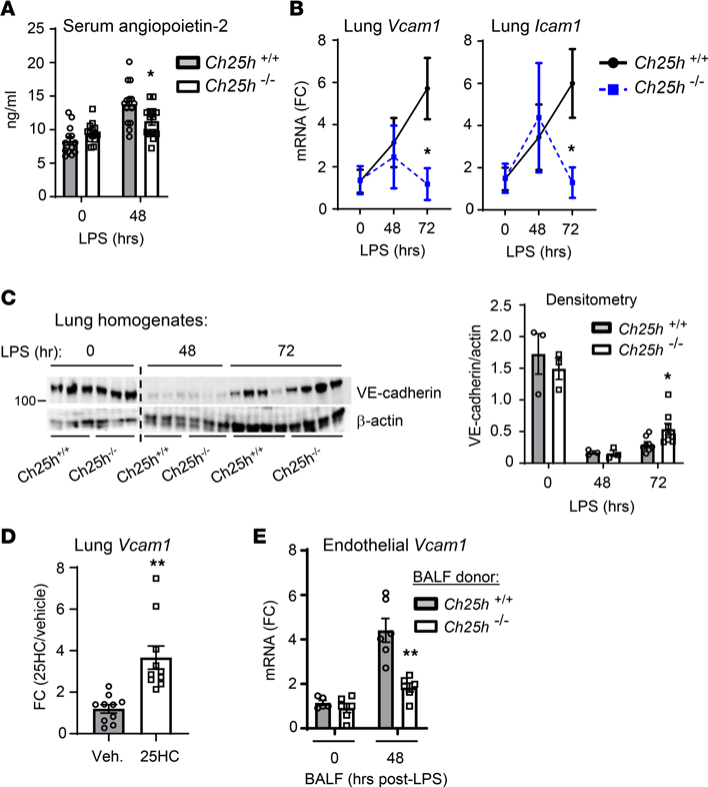
25HC induces endothelial activation and injury. (**A**) Serum angiopoietin-2 was measured in mice at the indicated time points relative to high-dose LPS aerosol inhalation (*n* = 13–14 per genotype). (**B**) Lung tissue from *Ch25h^+/+^* and *Ch25h^–/–^* mice was analyzed by quantitative PCR (qPCR) for the targets shown following LPS inhalation (*n* = 4–5 per genotype). (**C**) Lung homogenates were evaluated by immunoblot for VE-cadherin and β-actin (loading control) in *Ch25h^+/+^* and *Ch25h^–/–^* mice at baseline and 48 hours or 72 hours after high-dose LPS aerosol inhalation. Immunoblot results from independent mice are shown in the left panel, and densitometry for actin-normalized VE-cadherin signal in the right panel (*n* = 3–8 per condition). The dashed line indicates juxtaposition of nonadjacent portions of the original gel. (**D**) Lung tissue from WT mice was analyzed by qPCR for *Vcam1* following i.p. treatment with 25HC or vehicle (*n* = 10 per treatment). (**E**) Mouse pulmonary microvascular endothelial cells were cultured for 24 hours in 50% medium/50% BALF (0 or 48 hours after inhaled LPS) collected from mice of the indicated genotype and then analyzed by qPCR for *Vcam1*. *P < 0.05, **P < 0.01 by unpaired 2-tailed *t* test. FC, fold change.

Consistent with attenuated endothelial injury in LPS-exposed *Ch25h^–/–^* mice, we found that serum levels of angiopoietin-2, a marker of endothelial injury (22), were lower than in WT counterparts (Figure 2A). In addition, induction of vascular cell adhesion molecule 1 (*Vcam1*) and intercellular adhesion molecule 1 (*Icam1*) was less sustained in *Ch25h^–/–^* lung homogenates, returning to baseline by 72 hours after LPS (Figure 2B). VE-cadherin signal in lung homogenates exhibited similar kinetics, degrading equivalently up to 48 hours after LPS in *Ch25h^+/+^* and *Ch25h^–/–^* mice, denoting comparable initial endothelial cell adherens junction injury, but then recovering more rapidly in *Ch25h^–/–^* mice by 72 hours (Figure 2C). Systemically delivered (i.p.) 25HC robustly induced *Vcam1* in the lungs of naive WT mice (Figure 2D) but did not increase BALF protein (not shown), suggesting that 25HC is sufficient to activate the pulmonary endothelium in vivo but not sufficient, at least with this dosing regimen, to cause pulmonary vascular leak. In order to model the milieu of the CH25H-sufficient lung, BALF was collected from *Ch25h^+/+^* and *Ch25h^–/–^* mice 48 hours after LPS and applied to murine pulmonary microvascular endothelial cells in culture. *Ch25h^+/+^* BALF robustly induced endothelial *Vcam1*, whereas *Ch25h^–/–^* BALF did not (Figure 2E).

**Figure 3:**
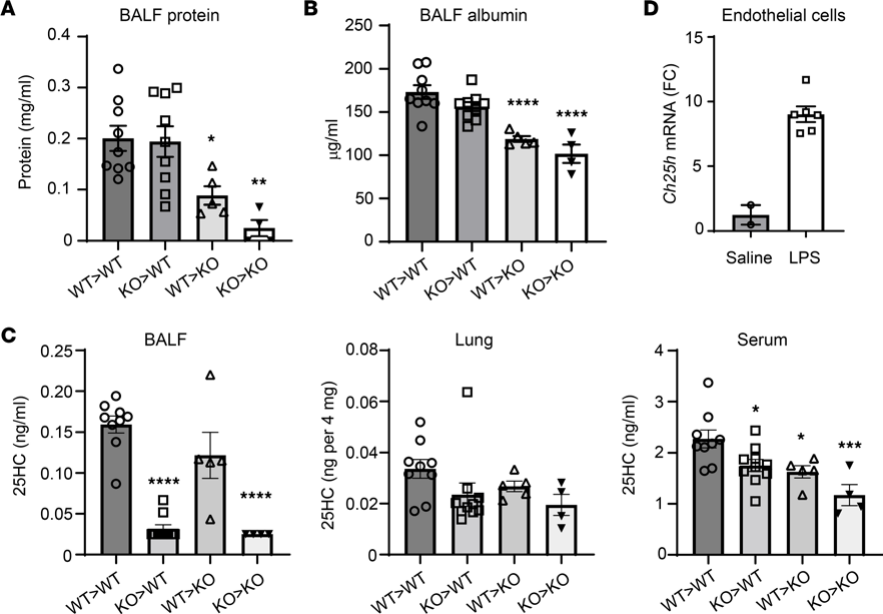
*Ch25h* in non-hematopoietic cells regulates lung injury. (**A**–**C**) Mice chimeric for *Ch25h* expression in hematopoietic and/or non-hematopoietic cells were produced by bone marrow transfer from *Ch25h^+/+^* (WT) or *Ch25h^–/–^* (KO) mice into WT or KO recipients following sublethal irradiation (graphs show donor > recipient). Chimeras were then exposed to inhaled LPS. At 72 hours after exposure, protein (**A**) and albumin (**B**) were measured in BALF, and 25HC was measured in BALF, lung, or serum (**C**) (*n* = 4–10 per condition, representative of 2 independent experiments). (**D**) Endothelial cells (CD45^–^EpCAM^–^CD31^+^) were FACS-sorted from mouse lung 72 hours after saline or LPS inhalation. RNA was prepared and qPCR performed for *Ch25h* (*n* = 2 saline, *n* = 6 LPS). In **A**–**C**, all chimeras with statistically significant differences in 25HC compared with WT>WT are indicated with asterisks. *P < 0.05, **P < 0.01, ***P < 0.001, ****P < 0.0001 compared with WT>WT by 1-way ANOVA with Dunnett’s post hoc test. FC, fold change.

*Ch25h* is reportedly expressed not only in hematopoietic cells such as AMs, but also in endothelial and epithelial cells (23, 24). In order to investigate whether hematopoietic or non-hematopoietic cell CH25H drives vascular leak in the LPS-exposed lung, we generated bone marrow chimeric mice, transferring *Ch25h^+/+^* or *Ch25h^–/–^* bone marrow into irradiated *Ch25h^+/+^* or *Ch25h^–/–^* recipients (Supplemental Figure 5). Interestingly, we found that *Ch25h* deletion in non-hematopoietic cells was sufficient to reduce markers of microvascular injury in LPS-exposed mice (Figure 3, A and B). Deletion of *Ch25h* in hematopoietic cells was not sufficient to reduce injury but appeared to augment protection on a background of non-hematopoietic *Ch25h* deletion (i.e., KO>KO compared with WT>KO chimeras). For confirmation, 25HC was measured by liquid chromatography–mass spectrometry in BALF, lung (perfused and lavaged), and serum of the chimeras. As is evident in Figure 3C, BALF 25HC tracked with hematopoietic cell CH25H, whereas both hematopoietic and non-hematopoietic cell CH25H appeared to contribute to serum 25HC. Modest, statistically non-significant reductions in lung 25HC were observed in chimeras with hematopoietic and non-hematopoietic cell CH25H deficiency. In line with our prior finding that inhaled LPS increases serum 25HC in mice (2) and supporting a possible role specifically for endothelial CH25H in the vascular leak phenotype, we found that inhaled LPS upregulated *Ch25h* in endothelial (CD45^–^EpCAM^–^CD31^+^) cells sorted from WT mouse lung (Figure 3D and Supplemental Figure 6).

**Figure 4:**
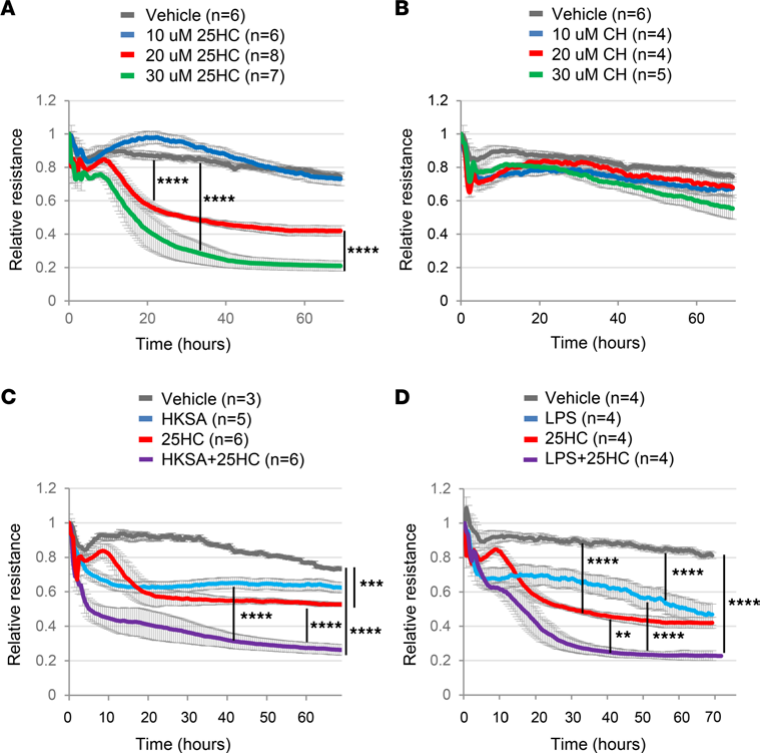
25HC decreases transendothelial resistance. (**A** and **B**) Human pulmonary artery endothelial cells (HPAECs) were cultured in medium with 2% FBS supplemented with vehicle or the indicated concentrations of 25HC (**A**) or cholesterol (CH; a negative control) (**B**), and transendothelial electrical resistance (TER) measurements were performed over a 70-hour time period. (**C**) HPAECs were incubated with vehicle or heat-killed *S*. *aureus* (HKSA; 2 × 10^8^ particles/mL) for 30 minutes followed by treatment with vehicle or 25HC (20 μM) and TER monitored over 70 hours. (**D**) HPAECs were incubated with vehicle or LPS (100 ng/mL) for 30 minutes followed by treatment with vehicle or 25HC (20 μM) and TER monitored for 70 hours. Data are mean ± SEM and are representative of 2–3 independent experiments. The number of replicates per condition is given in the figure. Intercurve differences at 60 hours were analyzed by 1-way ANOVA with all pairwise post hoc comparisons and Tukey’s adjustment. **P < 0.01, ***P < 0.001, ****P < 0.0001.

Confirming that 25HC can compromise endothelial barrier integrity, we found that, in vitro, 25HC but not cholesterol was sufficient to decrease transendothelial electrical resistance (i.e., to increase paracellular leak across an endothelial monolayer) and that it did so in a concentration-dependent manner (Figure 4, A and B). Moreover, exogenous 25HC exacerbated the effects of both heat-killed *Staphylococcus* *aureus* (*S*. *aureus*) and LPS on electrical resistance (Figure 4, C and D). Taken together, these findings suggest that 25HC is sufficient to activate the endothelium and compromise pulmonary endothelial barrier integrity, but that it may also do so cooperatively with pathogen- or LPS-induced signals such as cytokines (which are increased in the CH25H-sufficient lung; Figure 1E).

Suggesting that 25HC may compromise endothelial barrier integrity by disassembling cell adherens junctions, we found that 25HC but not cholesterol caused downregulation and disorganization of VE-cadherin signal in human pulmonary artery endothelial cell monolayers (Supplemental Figure 7). Moreover, 25HC but not cholesterol increased endothelial cell monolayer permeability to FITC-avidin and also exacerbated permeability increases induced by heat-killed *S*. *aureus* (Supplemental Figure 8).

### 25HC induces ER stress in the pulmonary endothelium.

**Figure 5:**
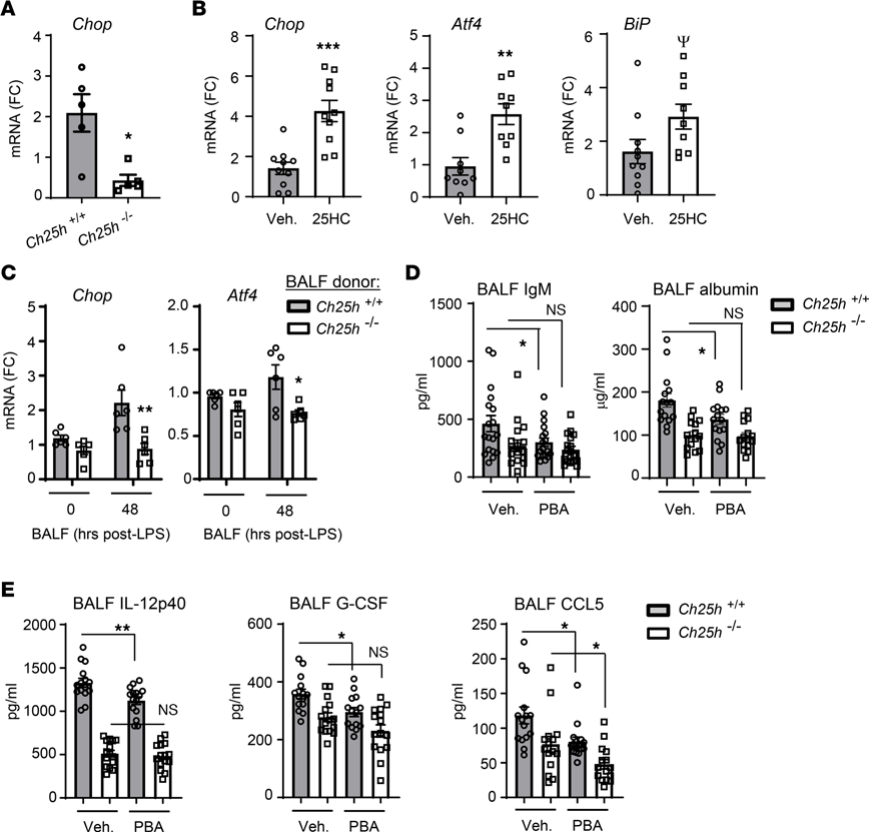
CH25H/25HC induces ER stress in the injured lung. (**A**) Lung tissue from *Ch25h^+/+^* and *Ch25h^–/–^* mice was analyzed by qPCR for *Chop* 72 hours after LPS inhalation (*n* = 10 per genotype). (**B**) Lung tissue from WT mice was analyzed by qPCR for the indicated targets after i.p. treatment with 25HC or vehicle (*n* = 8–10 per condition). (**C**) Mouse pulmonary microvascular endothelial cells were cultured for 24 hours in 50% medium/50% BALF (0 or 48 hours after inhaled LPS) collected from mice of the indicated genotypes and then analyzed by qPCR for the indicated targets. (**D**) Mice of the indicated genotypes were treated with 100 mg/kg 4-phenylbutyric acid (PBA) or PBS vehicle (Veh.) i.p. at –1, +6, and +24 hours in relation to LPS inhalation and then had BALF IgM and albumin quantified at 72 hours after LPS (*n* = 15–18 per condition). (**E**) Mice were treated as in **D** and then had the indicated cytokines quantified in BALF (*n* = 15 per condition). Data are mean ± SEM and are representative of 2–3 independent experiments. ^Ψ^*P* = 0.06, *P < 0.05, **P < 0.01, ***P < 0.001 by unpaired 2-tailed *t* test. FC, fold change.

25HC has been reported to cause cytotoxicity by inducing ER stress (20, 21). Given this, we hypothesized that endogenous CH25H-derived 25HC might compromise endothelial barrier functions through inducing ER stress. Compared with WT counterparts, *Ch25h^–/–^* lungs had markedly lower expression of the ER stress gene *Chop* (i.e., *Ddit3*) following LPS exposure (Figure 5A). Conversely, i.p. 25HC upregulated *Chop* and the additional ER stress markers *BiP* and *Atf4* in the lungs of naive WT mice (Figure 5B). ER stress markers were higher in the lungs of naive LXR-null mice than in naive WT counterparts, suggesting that induction of ER stress by 25HC is LXR independent (Supplemental Figure 9). We found that 48 hours after LPS, BALF from *Ch25h^+/+^* but not *Ch25h^–/–^* mice induced *Chop* and *Atf4* when applied to murine pulmonary microvascular endothelial cells in culture (Figure 5C), again suggesting that the soluble mediators induced in the *Ch25h^–/–^* airway are less injurious to the endothelium than are those induced in the WT airway. Suggesting that the higher ER stress in *Ch25h^+/+^* as compared with *Ch25h^–/–^* lungs drives their increased vascular leak, we found that systemic treatment with the ER stress–relieving agent 4-phenylbutyric acid (PBA) (25) significantly reduced BALF IgM and albumin in *Ch25h^+/+^* but not *Ch25h^–/–^* mice exposed to inhaled LPS, essentially equalizing the degree of vascular leak between the 2 genotypes (Figure 5D). PBA also partially reduced BALF IL-12p40 and G-CSF in *Ch25h^+/+^* but not *Ch25h^–/–^* mice, whereas it reduced BALF CCL5 in mice of both genotypes (Figure 5E).

We next sought to determine whether CH25H had a correlation to ER stress in human ARDS. As patient lung biopsies were not available, we examined expression of ER stress genes in AMs collected from patients by bronchoalveolar lavage. Specimens analyzed were from patients (*n* = 30) enrolled within 48 hours of disease onset at 5 North American medical centers in a phase II placebo-controlled trial of omega-3 fatty acids for the treatment of ARDS (Supplemental Table 1) (2, 26). Strong correlations of AM *CH25H* were indeed found to AM expression of *BIP*, *ATF6*, and *XBP1* (Supplemental Figure 10), suggesting that 25HC may also be involved in promoting augmented ER stress in the injured human lung.

### AM CH25H and 25HC track with severity in human ARDS.

With augmented lung injury emerging as a dominant phenotype for CH25H in a high-dose LPS exposure model and our prior finding that LPS also induces AM *CH25H* and BALF 25HC in humans (2), we speculated that levels of the enzyme and its product might track with alveolar capillary leak and disease severity in patients with ARDS. To address this, we next analyzed AM *CH25H* expression (*n* = 30) and BALF 25HC concentration (*n* = 81) in relation to vascular and clinical measures in patients with ARDS (Supplemental Tables 1 and 2) (2, 26).

**Figure 6:**
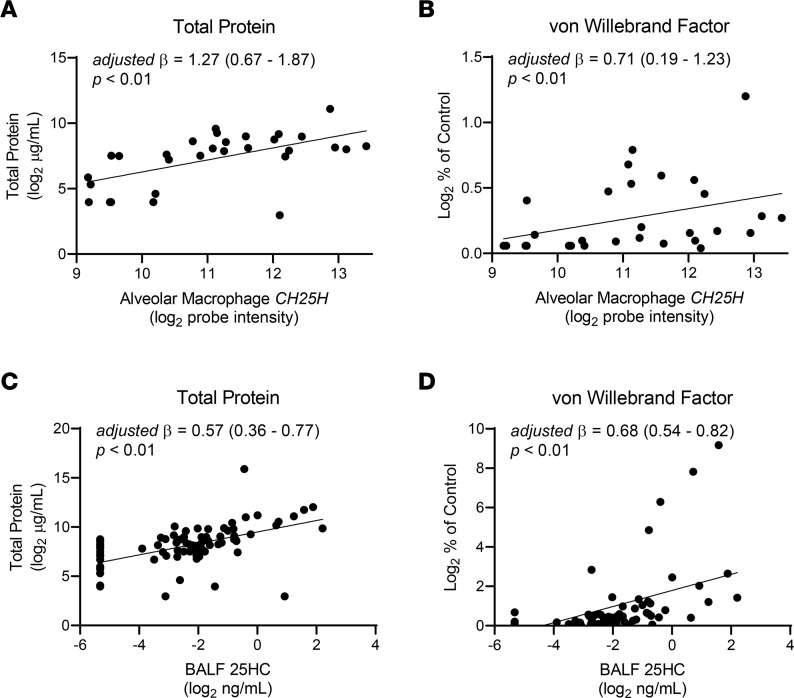
AM *CH25H* expression and 25HC BALF concentrations are associated with higher BALF total protein and von Willebrand factor levels. AM *CH25H* mRNA (quantified by microarray) (*n* = 30) and BALF 25HC levels (quantified by mass spectrometry) (*n* = 81) were measured in patients within 48 hours of ARDS diagnosis from a therapeutic trial of omega-3 fatty acids. Depicted are the individual values and linear regression lines showing associations between AM *CH25H* expression and (**A**) total BALF protein and (**B**) von Willebrand factor levels, as well as between BALF 25HC levels and (**C**) total BALF protein and (**D**) von Willebrand factor levels. The estimates and *P* values are adjusted for age, sex, treatment group, and APACHE II severity of illness scores.

**Figure 7:**
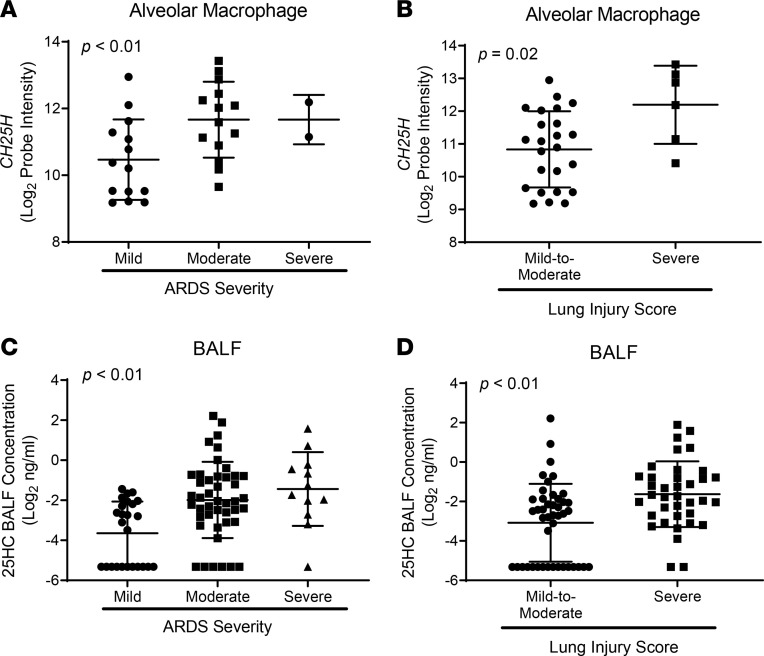
AM *CH25H* and airspace 25HC track with lung inflammation and injury in human ARDS. AM *CH25H* mRNA (quantified by microarray) (*n* = 30) and BALF 25HC (quantified by mass spectrometry) (*n* = 81) were measured in patients within 48 hours of ARDS diagnosis from a therapeutic trial of omega-3 fatty acids. (**A** and **C**) Multiple linear regression, adjusted for age, sex, APACHE II score, and treatment group, was used to test the relationship between normalized log_2_ *CH25H* probe intensity (**A**) or log_2_ BALF 25HC concentration (**C**) and the Berlin definition of ARDS severity as assessed by P_a_O_2_/F_I_O_2_ (P/F) ratio (P/F = 200–300: mild; P/F = 100–200: moderate; P/F < 100: severe). (**B** and **D**) A *t* test was used to compare normalized log_2_ AM *CH25H* probe intensity (**B**) or log_2_ 25HC BALF concentration (**D**) in patients with mild-to-moderate versus severe ARDS as assessed by the Murray Lung Injury Score (LIS) definition (LIS = 0.1–2.5: mild-to-moderate; LIS > 2.5: severe). Depicted are the individual patient values, mean, and SD.

First, examining the relationship of *CH25H* and 25HC to biologic measures of lung injury by linear regression, we found that increases in AM *CH25H* expression and 25HC BALF concentration were both associated with increased BALF levels of total protein and von Willebrand factor, a marker of endothelial injury and activation (Figure 6) (27). These associations persisted after adjustment for illness severity (APACHE II score), age, sex, and treatment group. Increased AM *CH25H* and BALF 25HC were also associated with heightened clinical severity in ARDS patients, as measured by decreased P_a_O_2_/F_I_O_2_ ratio, a clinical metric of oxygenation (Figure 7, A and C), and increased Lung Injury Score, a multiparameter score involving chest imaging and lung function measures (Figure 7, B and D) (28). Correlation analyses of AM *CH25H* and 25HC in relation to P_a_O_2_/F_I_O_2_ ratio further supported the strength of these relationships (Supplemental Figure 11). Consistent with our in vivo mouse data demonstrating associations between *Ch25h* and inflammatory cytokines/chemokines (Figure 1E), we also found that higher AM *CH25H* expression and 25HC BALF concentrations were associated with increased BALF levels of IL-8, IL-6, and IL-17A in ARDS patients (Supplemental Table 3). Collectively, these findings from human ARDS closely parallel those in the LPS mouse lung injury model, in which higher cytokines and microvascular injury were seen in *Ch25h^+/+^* mice than in 25HC-deficient counterparts.

## Discussion

We recently reported that *Ch25h* is induced in monocyte-derived AMs recruited to the lung days after LPS inhalation, and that CH25H-derived 25HC activates LXR in these macrophages to drive pro-resolving phagocytic clearance of apoptotic cells (efferocytosis) (2). *Ch25h* is, however, also robustly upregulated in lung-resident cells within 2–8 hours of LPS inhalation, inducing ng/mL-range 25HC in the airspace and serum (2). 25HC induction is completely dependent on CH25H, as it is not observed in *Ch25h^–/–^* mice. In the present report, we questioned whether CH25H upregulation in lung-resident cells also regulates the induction phase of lung inflammation and whether induction-phase 25HC acts on lung-resident cells to impact lung injury. To test this, we evaluated *Ch25h^–/–^* mice in a high-dose LPS model of severe lung injury.

Consistent with a prior report that 25HC amplifies proinflammatory cytokines by increasing AP-1 binding at gene promoters (7), we found sustained reductions in cytokine levels in the BALF of *Ch25h^–/–^* mice. In addition to this, microvascular leak, a hallmark of lung injury, was substantially reduced in *Ch25h^–/–^* mice, along with markers of endothelial activation, whereas this was not observed in mice deleted for LXR, a well-established nuclear receptor for 25HC and other oxysterols (1). These findings suggest that native 25HC, directly and/or indirectly (e.g., through augmentation of cytokine levels), and via an LXR-independent mechanism, activates the endothelium and compromises endothelial barrier integrity. Supporting a direct effect, we found that treatment of naive mice with systemically delivered 25HC induced lung *Vcam1*. In addition, exogenous 25HC was sufficient to promote increased permeability across an endothelial monolayer, and lung injury was found to track with non-hematopoietic cell *Ch25h*. However, our finding that systemic treatment of naive WT mice with i.p. 25HC was not sufficient to induce vascular leak (increased BALF protein) in the lung suggests that, in vivo, 25HC may require a background proinflammatory cytokine milieu or other concurrent signals to induce pro-injury effects.

We propose that the ER membrane, and, in particular, the ER stress response, may possibly serve as an integrator and tipping point that determines opposing LXR-dependent versus -independent effects of 25HC on inflammation. 25HC reportedly causes LXR-independent ER stress (21), and ER stress is reported both to activate AP-1 and NF-κB (29) and to sustain late-phase cytokine induction by LPS (16). By contrast, LXR activation protects against ER stress and associated inflammation by remodeling ER membrane phospholipid (30), a finding consistent with our observation of elevated ER stress markers in the naive LXR-null lung. We speculate that the ER-resident oxysterol-binding protein–related protein 8 may possibly regulate this balance as it promotes 25HC-induced ER stress and appears to restrict 25HC bioavailability to LXR (31, 32). Although 25HC is released extracellularly and may thus act paracellularly, our findings that *Ch25h* is robustly induced in pulmonary endothelial cells and that non-hematopoietic rather than hematopoietic cell *Ch25h* is the primary driver of vascular leak together suggest that an endothelial CH25H/25HC circuit may act locally within pulmonary endothelial cells. Generation of endothelial specific *Ch25h*-null mice will be required to resolve this question, and future investigations with extrapulmonary/vascular models of acute lung injury (e.g., sepsis) may also be revealing. Indeed, our in vivo findings, including those derived from the chimeras, do not exclude the possibility of contributions from epithelial CH25H. In addition, PBA, like other chemicals, is imperfectly selective for ER stress and nonselective for endothelial cells. Thus, future studies using additional pharmacologic or genetic interventions on ER stress are warranted. We also acknowledge that our in vitro studies of endothelial cells with supplemental 25HC do not probe the role of native CH25H/25HC in endothelial permeability.

The finding that AM *CH25H* and BALF 25HC are associated with disease severity and markers of endothelial activation and injury in ARDS patients suggests that 25HC may mediate lung injury and inflammation through similar mechanisms in humans and mice. Unlike in mice, our data do not demonstrate a functional role for 25HC in human lung disease. It is possible that 25HC may simply mark those patients who have more severe lung inflammation. Patients with chronic obstructive pulmonary disease were recently also shown to have increased sputum 25HC (33), suggesting that 25HC may be a biomarker of airway inflammation across lung diseases. Nonetheless, in our study, the association between AM *CH25H* gene expression and alveolar total protein, even after adjustment for severity of illness, lends some plausibility that this pathway may play a pathophysiologic role in the alveolar capillary leak that is the hallmark of ARDS.

Interesting comparisons may be drawn between our findings regarding the effect of 25HC on the pulmonary endothelial barrier and those described in the literature regarding the effect of oxidized phospholipid species. Full-length oxidized 1-palmitoyl-2-arachidonoyl-*sn*-glycero-3-phosphorylcholine (oxPAPC) has well-described protective and antiinflammatory activities upon the endothelium, reducing vascular leak induced by diverse exposures through mechanisms including NF-κB inhibition, cAMP elevation, and induction of heme oxygenase-1 and lipoxin A4 (34). By contrast, truncated oxPAPC species, which rise in the inflamed lung (35), induce endothelial barrier disruption and amplify inflammation (36). It is thought that the presence of truncated species within the total oxPAPC pool may explain the observed dose dependence of oxidized phospholipids on endothelial barrier integrity, with protection at low concentrations and disruption at high concentrations (34). We also found that the impact of CH25H-derived 25HC on the lung is dependent on exposure intensity, with prominent pro-resolving effects during mild inflammation (2), but pro-injury effects during severe inflammation (the present report). While we do not have reason to suspect increased levels of structurally modified (e.g., secondarily oxidized) 25HC derivatives in the latter situation, it is possible that the thresholds for pro-resolving (LXR activation) versus pro-injury (ER stress) activities of 25HC are concentration and/or milieu dependent.

In closing, CH25H exerts dual roles in lung tissue homeostasis during low- versus high-injury states that we propose to arise from differing cellular origins and molecular targets of 25HC in these 2 contexts (Supplemental Figure 12). After low-dose LPS inhalation, a model of airspace inflammation with minimal vascular injury, AM-derived 25HC has the predominant effect of activating LXR-dependent macrophage efferocytosis, thereby promoting resolution of airway neutrophilia (2). By contrast, after high-dose LPS, a model of severe acute lung injury, we posit that endothelial derived 25HC locally activates the endothelium, potentially through LXR-independent ER stress, thereby inducing adhesion molecules and cytokines and causing adherens junction assembly. This has the predominant impact of exacerbating vascular leak. These dual roles may suggest that pharmacologic targeting of CH25H in human disease is likely to prove challenging. Nonetheless, the mixed roles of this lipid in lung health and disease suggest that future studies are warranted to better define its molecular targets and functions in the lung and perhaps also to identify and pursue clinical contexts in which its manipulation may possibly be used to therapeutic benefit.

## Methods

### Reagents.

*Escherichia coli* 0111:B4 LPS, penicillin, and streptomycin were from MilliporeSigma. *K*. *pneumoniae* 43816 (serotype 2), DMEM, and FBS were from American Type Culture Collection. 25HC and cholesterol were purchased from MilliporeSigma. The Bio-Rad protein assay was used (catalog 5000006).

### Mice.

Female and male mice, 7–10 weeks old and weighing 18–22 g, were used. *Ch25h^–/–^* mice were produced as previously described (3). C57BL/6 mice were from The Jackson Laboratory. *Nr1h2^–/–^* and *Nr1h3^–/–^* mice were provided by David Mangelsdorf (University of Texas Southwestern Medical Center, Dallas, Texas, USA) and were intercrossed to produce *Nr1h2^–/–^* *Nr1h3^–/–^* mice. All aforementioned strains were at least 8 generations backcrossed to C57BL/6. In selected experiments, littermate controls were used. For other experiments, C57BL/6 controls were used.

### In vivo murine exposures.

Exposure to aerosolized LPS (3 mg/mL, 30 minutes) was as previously described (15). *K*. *pneumoniae* (2,000 CFU/50 μL) was delivered to lung by oropharyngeal aspiration during isoflurane anesthesia (14). In some studies, mice were treated i.p. with 50 mg/kg 25HC or vehicle (hydroxypropyl beta cyclodextrin, Cyclo Therapeutics Inc.) daily for 3 days with sacrifice 4 hours after the final dose. In other experiments, mice were treated with 100 mg/kg 4-phenylbutyric acid (MilliporeSigma) or PBS control i.p. at –1, +6, and +24 hours in relation to LPS inhalation (16).

### Generation of bone marrow chimeric mice.

To generate bone marrow chimeric mice, procedures were followed as previously described (37), with minor modifications. *Ch25h^+/+^* mice that were congenic for CD45 (stock 002014, The Jackson Laboratory) were used. Recipients were lethally irradiated (11 Gy) by a model 431 irradiator using a ^137^Cs source (JL Shepherd and Associates). Within about 4 hours of irradiation, donor-derived bone marrow (about 2 × 10^6^ to 5 × 10^6^ cells) was injected i.v. into recipients. The efficiency of donor stem cell engraftment was determined by flow cytometry for CD45.1 (*Ch25h^+/+^*) as shown in Supplemental Figure 5. Engraftment efficiency within all experimental animals tested was greater than 97.5%. Chimeras were used a minimum of 10–11 weeks after transplant.

### Acute lung injury/ARDS patient cohort studies.

The BALF samples analyzed were previously collected from subjects enrolled at 5 North American centers for the Phase II Randomized Placebo-Controlled Trial of Omega-3 Fatty Acids for the Treatment of Acute Lung Injury trial, conducted from 2006 to 2008 (26, 38). Patients were enrolled into the trial within 48 hours of ARDS onset. All samples used for our analysis were obtained from study subjects before their receipt of placebo or treatment. For a subset of patients, AMs were purified by negative selection with antibody-labeled microbeads for CD3, CD15, CD19, CD235a, CD294, and CD326. RNA was extracted from AMs and hybridized to an Illumina HumanRef-8 Beadchip inclusive of 18,415 genes. Raw microarray data were processed for variance stabilization and quantile normalization using the Bioconductor package lumi (39). The data were deposited in the NCBI’s Gene Expression Omnibus database under accession number GSE89953.

### Murine BALF collection and analysis.

BALF was collected immediately after sacrifice and cell counts performed, as previously described (15). Total protein was quantified by the method of Bradford (14). Bacterial colony-forming units were quantified in BALF and blood using previously reported methods (37).

### Mouse lung endothelial cell studies.

Mouse primary lung microvascular endothelial cells (Cell Biologics C57-6011) were cultured in endothelial cell growth medium (Cell Biologics M1168) and allowed to grow for 2 days past confluence to form a monolayer. The medium was then changed in triplicate wells to 50% growth medium/50% pooled BALF (*n* = 3 mice) from WT or *Ch25h^–/–^* mice collected 0 or 48 hours after LPS inhalation. Following an incubation period of 24 hours, RNA was obtained using RNeasy Plus Mini Kit (Qiagen 74134).

### Protein analyses.

Cytokines were quantified in mice by multiplex assay (Bio-Plex, Bio-Rad). IL-8 and IL-6 were quantified in human BALF using a Luminex bead-based immunoassay (R&D Systems Inc.), and IL-17A by a chemiluminescence immunoassay (Meso Scale Discovery). Total protein concentration was measured in human BALF using the bicinchoninic acid protein assay (Thermo Fisher Scientific). ELISAs for albumin and IgM were from Bethyl Laboratories and for angiopoietin-2 from R&D Systems Inc., and were performed per manufacturer instructions. Von Willebrand factor (vWF) was measured using an ELISA (Diagnostica Stago) with the results expressed as a percentage of the vWF control reference provided with the kit. The vWF control reference has been assayed against a secondary standard of the International Standard of vWF.

### RNA isolation and quantitative PCR.

RNA was isolated by RNeasy Kit (Qiagen). Complementary DNAs were generated from 1.5 μg of purified RNA using TaqMan reverse transcription reagents from Applied Biosystems. Real-time PCR was performed in triplicate with TaqMan PCR Mix (Applied Biosystems) in the HT7900 ABI sequence Detection System (Applied Biosystems). Predesigned primers were purchased from Applied Biosystems (*Vcam1* [Mm01320970_m1], *Atf4* [Mm00515325_g1], *Ddit3* [Mm01135937_g1], *Ch25h* [Mm00515486_s1], and *Icam1* [Mm00516023_m1]), using *Gusb* (Mm00446953_s1) or *Gapdh* (Mm99999915_g1) as a housekeeping gene for normalization.

### Endothelial cell sorting and permeability assays.

Sorting of murine pulmonary endothelial cells was as previously described (40). Briefly, lungs were inflated with digestion buffer (RPMI plus HEPES; elastase [Worthington Biochemical]; dispase; and DNase I [MilliporeSigma]), removed, and incubated in digestion buffer (45 minutes, 37°C). After digestion, the lobes were dissected from the trachea, diced, filtered through a 70 μm strainer, and further digested for 30 minutes in RPMI plus HEPES, liberase (Roche), and DNase I. RBCs were lysed with Türk’s lysing buffer, and digested cells were stained with anti-CD45–PE (clone 30-F11), anti-CD31–eF450 (clone 390), anti-EpCAM–APC (clone G8.8), and 7AAD (MilliporeSigma). Anti-CD45–biotin (BioLegend) and anti-Ter119–biotin (BioLegend) were added to each sample, and EasySep Mouse Streptavidin Rapid Spheres with an EasySep magnet (STEMCELL Technologies) were used for removal of CD45^+^ cells and RBCs prior to sorting on a FACS ARIA-II (BD Biosciences). Transendothelial electrical resistance was measured across confluent human pulmonary artery endothelial monolayers (passage 4–6; Lonza) in medium containing 2% FBS, using an electrical cell-substrate impedance sensing system (Applied Biophysics), as previously described (35).

### Histopathologic analysis.

Tissues were fixed in 10% neutral-buffered formalin, trimmed, processed for paraffin, embedded, sectioned (5 μm), and stained with H&E. Slides were scanned using an Aperio ScanScope XT Scanner (Aperio Technologies Inc.). Lung histopathology following LPS inhalation was scored in a blinded fashion by a board-certified veterinary pathologist, as previously described (41).

### Immunoblotting.

Lungs were homogenized in 1× PBS plus 0.1% Triton X-100 supplemented with HALT protease and phosphatase inhibitor (Thermo Fisher Scientific), using a TissueLyser (Qiagen). Equal protein mass was run on a 10% SDS-polyacrylamide gel and transferred to a PVDF membrane using standard methods. The membrane was probed with rabbit anti–VE-cadherin (1:1,000; Abcam) and anti–β-actin–HRP (1:10,000; MilliporeSigma). Membranes were then washed and exposed (60 minutes) to 1:5,000 HRP-conjugated secondary antibody (GE Healthcare) in 5% milk/buffer. After further washes, signal was detected with ECL Western Blot detection reagents (GE Healthcare), followed by film exposure (GE Healthcare).

### FACS analysis.

Cells isolated from BAL were pelleted in 1.5 mL microcentrifuge tubes and resuspended in FACS buffer (PBS supplemented with 1% BSA and 0.1% sodium azide). Alternatively, in analyses of PMN infiltration into lung parenchyma, mice received an i.v. injection of anti-CD45 antibody about 10 minutes before sacrifice, and then at time of sacrifice blood was collected, residual blood perfused from lungs with 10 mL of saline, and lungs excised for digest. Fc receptor binding was blocked with 0.25 μg purified anti–mouse CD16/32 antibody specific for FcγRIII/II. Up to 1 × 10^6^ cells were stained for 30 minutes on ice in 50 μL of FACS buffer with the following dyes and antibodies: LIVE/DEAD Fixable Blue Dead Cell Stain Kit (Invitrogen), Ly6C (FITC), Ly6G (APC), CD45.2 (APC-Cy7), MerTK (PE), CD11c (PE-Cy7), CD64 (PerCP-Cy5.5), CD45.1 (Pacific Blue), Siglec-F (BV650), CD115 (BV711). Afterward, cellular events were acquired using a BD LSR-II Fortessa instrument and were analyzed using FlowJo 9.9.6 software.

### Mass spectrometric quantitation of oxysterols.

Sterols and oxysterols were quantified as previously described (3). Briefly, BALF and plasma were extracted with organic solvents and sterols and oxysterols quantified using deuterated and primary standards on an API-5000 Triple Quadrupole liquid chromatography mass spectrometer (SCIEX) (42).

### Permeability visualization assay.

Endothelial permeability to macromolecules was determined by express permeability testing (XPerT) assay developed by our group (43). This assay is based on high-affinity binding of cell-impermeable, avidin-conjugated, FITC-labeled tracer to the biotinylated extracellular matrix proteins immobilized on the surface covered with endothelial cell monolayers. Briefly, after cell stimulation with agonists, FITC-avidin solution was added to the culture medium for 3 minutes before termination of the experiment. Unbound FITC-avidin was washed out with PBS (pH 7.4, 37°C), cells were fixed with 3.7% formaldehyde in PBS (10 minutes, room temperature), and visualization of FITC-avidin on the bottoms of coverslips was performed using Nikon imaging system Eclipse TE 300 equipped with a digital camera (DKC 5000, Sony); ×10 objective lenses were used. Images were processed with Photoshop 7.0 software (Adobe Systems).

### Immunofluorescence staining and image analysis.

After experimental treatments, cells were fixed in 3.7% formaldehyde solution in PBS for 10 minutes at 4°C, washed with PBS, permeabilized with 0.1% Triton X-100 in PBS for 30 minutes at room temperature, and blocked with 2% BSA in PBS for 30 minutes. Incubation with VE-cadherin antibody and DAPI was performed for 1 hour at room temperature followed by staining with Alexa Fluor 488–conjugated secondary antibody. After immunostaining, the slides were analyzed using an inverted microscope Nikon Eclipse TE300 connected to SPOT RT monochrome digital camera and image processor (Diagnostic Instruments). The images were processed with Adobe Photoshop 7.0 (Adobe Systems). For each experimental condition, at least 10 microscopic fields in each independent experiment were analyzed.

### Wet-to-dry lung mass analysis.

Lungs were necropsied following sacrifice, trimmed, and promptly weighed (wet mass). The lungs were then fully desiccated under vacuum and reweighed (dry mass). Wet/dry lung mass was represented as a ratio.

### Statistics.

For murine cell and animal studies, analysis was performed using GraphPad Prism software. Data are represented as mean ± SEM. Two-tailed Student’s *t* test was applied for comparisons of 2 groups, and 1-way ANOVA for comparisons of more than 2 groups. For the patient studies, total protein concentration, cytokine concentration, 25HC concentration, and microarray probe intensities were analyzed as continuous variables after log_2_ transformation given the distribution of the data. Eighteen of the 81 BALF specimens had a 25HC concentration below the lower limit of detection (LLOD) of 0.05 ng/mL. These specimens were assigned a 25HC concentration of one-half the LLOD. P_a_O_2_/F_I_O_2_ ratios (P/F ratios) were divided into mild, moderate, or severe based on the Berlin definition of ARDS (44). LIS was divided into mild-to-moderate versus severe ARDS based on the original clinical scoring system (28).

A Pearson’s test was used to identify correlations between AM *CH25H* log_2_ probe intensity and AM *BIP*, *ATF6*, *XBP1*, and *CHOP* log_2_ probe intensities. Multiple linear regression was used to test for an association between either AM *CH25H* log_2_ probe intensity or log_2_ 25HC BALF concentration and log_2_ total protein concentration, von Willebrand factor percentage of control, Berlin categories of P/F ratio, and log_2_ alveolar cytokine concentrations (IL-8, IL-6, and IL-17A). Adjustments were made for age, sex, APACHE II score, and treatment group in all regressions. These covariates were selected a priori. We used a 2-tailed *t* test to compare either AM *CH25H* log_2_ probe intensity or log_2_ 25HC BALF concentration and LIS (mild-to-moderate vs. severe). Analyses were conducted using R version 3.5.1. For all tests, *P* less than 0.05 was considered significant.

### Study approval.

All rodent experiments were performed in accordance with the Animal Welfare Act and the US Public Health Service Policy on Humane Care and Use of Laboratory Animals after approval by the Animal Care and Use Committee of the NIH National Institute of Environmental Health Sciences (Research Triangle Park, North Carolina, USA). In the fish oil ARDS trial, participants were enrolled at 5 North American centers (University of Vermont; University of Toronto; Harborview Medical Center and the University of Washington in Seattle, Washington; and St. Alphonsus Regional Medical Center in Boise, Idaho). Informed consent was obtained prior to enrollment; human subjects committees at each site approved the trial, as did a Data Safety Monitoring Board appointed by the NIH National Heart, Lung, and Blood Institute.

## Author contributions

MBF designed and conducted experiments, analyzed and interpreted the data, and contributed to writing of the manuscript. JHM, YL, KGB, AAB, JMM, AA, and PR conducted and analyzed experiments. EDM, CM, RDS, PWK, JGM, BMT, and MMW analyzed and interpreted the data and contributed to writing of the manuscript. JHM and EDM contributed equally.

## Supplementary Material

Supplemental data

## Version 1 02/23/2023

In-Press Preview

## Version 2 04/10/2023

Electronic publication
