# Supplementary material for: 25-Hydroxycholesterol exacerbates vascular leak during acute lung injury
Source: JCI Insight. 2023 Apr 10;8(7):e155448. doi: 10.1172/jci.insight.155448 (PMC10132150; doi:10.1172/jci.insight.155448)
Supplement: Supplemental data [file jciinsight-8-155448-s040.pdf]

25-hydroxycholesterol exacerbates vascular leak during acute lung injury

Jennifer H. Madenspacher[1*], Eric D. Morrell[2*], Jeffrey G. McDonald[3,4], Bonne M. Thompson[3], Yue Li[5], Konstantin G. Birukov[5], Anna A. Birukova[5], Renee D. Stapleton[6], Aidin Alejo[1], Peer Karmaus[1], Julie M. Meacham[1], Prashant Rai[1], Carmen Mikacenic[2], Mark M. Wurfel[2], Michael B. Fessler[1]

[1]Immunity, Inflammation and Disease Laboratory, National Institute of Environmental Health Sciences, NIH, Research Triangle Park, NC 27709
[2]Section of Pulmonary, Critical Care, and Sleep Medicine, Harborview Medical Center, Seattle, WA 98104
[3]Center for Human Nutrition, University of Texas Southwestern Medical Center, Dallas, TX 75390
[4]Department of Molecular Genetics, University of Texas Southwestern Medical Center, Dallas, TX 75390
[5]Department of Anesthesiology, University of Maryland School of Medicine, Baltimore, MD 21201
[6]Department of Medicine, Larner College of Medicine, University of Vermont, Burlington, VT 05405

*****Equal contribution.

SUPPLEMENTAL MATERIALS

Table S1. Fish Oil Trial AM Gene Expression Microarray Subjects

Characteristic at Enrollment	Omega-3 Fatty Acid (n = 30)
Patient age, mean ± SD	44 ± 17
Male patients, n (%)	18 (60%)
Caucasian, n (%)	26 (87%)
Comorbidities, n (%)	
Diabetes	4 (13%)
Cirrhosis	2 (7%)
ARDS risk factor, n (%)*	
Sepsis	17 (57%)
Trauma	14 (47%)
Pneumonia	9 (30%)
Other	4 (13%)
APACHE II, mean ± SD	21 ± 6
P/F Ratio, mean ± SD	199 ± 61
LIS, median, IQR	2.25, 2.00 – 2.50
VFDs, median, IQR	13, 0 - 23
28-d Mortality, n (%)	4, (13%)

APACHE = Acute Physiology and Chronic Health Evaluation; ARDS = acute respiratory distress syndrome; LIS = lung injury score, P/F ratio = PaO_2/FiO_2 ratio; VFDs = ventilator-free days; * Risk factors for ARDS are not mutually exclusive.

Table S2. Fish Oil Trial Subjects with BALF 25HC Measurement

Characteristic at Enrollment	Omega-3 Fatty Acid (n = 81)
Patient age, mean ± SD	50 ± 16
Male patients, n (%)	51 (64%)
Caucasian, n (%)	72 (89%)
Comorbidities, n (%)	
Diabetes	17 (21%)
Cirrhosis	6 (7%)
ARDS risk factor, n (%)*	
Sepsis	54 (67%)
Pneumonia	35 (43%)
Trauma	30 (37%)
Other	10 (12%)
APACHE II, mean ± SD	22 ± 7
P/F Ratio, mean ± SD	166 ± 59
LIS, median, IQR	2.5, 2.25 – 3.00
VFDs, median, IQR	14, 0 – 21
28-d Mortality, n (%)	14, (17%)

APACHE = Acute Physiology and Chronic Health Evaluation; ARDS = acute respiratory distress syndrome; LIS = lung injury score, P/F ratio = PaO_2/FiO_2 ratio; VFDs = ventilator-free days; * Risk factors for ARDS are not mutually exclusive.

Table S3. Alveolar CH25H/25HC is Associated with Higher Levels of Inflammatory Biomarkers in ARDS

	**Alveolar macrophage *CH25H***		**BALF 25HC**	
	***β* (95% CI)**	***p***	***β* (95% CI)**	***p***
BALF IL- 8	1.12 (0.20 – 2.04)	0.02	0.24 (-0.02 – 0.51)	0.07
BALF IL-6	1.64 (0.92 – 2.37)	< 0.01	0.95 (0.69 – 1.21)	< 0.01
BALF IL-17A	0.51 (-0.29 – 1.30)	0.20	0.76 (0.47 – 1.04)	< 0.01

Linear regression between alveolar macrophage *CH25H* $\log_2$ probe intensity or $\log_2$ 25HC BALF concentration (ng/mL) and $\log_2$ transformed BALF IL-8, IL-6, or IL-17A concentrations (pg/mL). All analyses were adjusted for age, sex, APACHE II score, and treatment group (*n* = 30 for AM *CH25H* analysis; *n* = 81 for BALF 25HC analysis).

ARDS = acute respiratory distress syndrome; BALF = bronchoalveolar lavage fluid; *CH25H* = cholesterol-25-hydroxylase; 25HC = 25-hydroxycholesterol

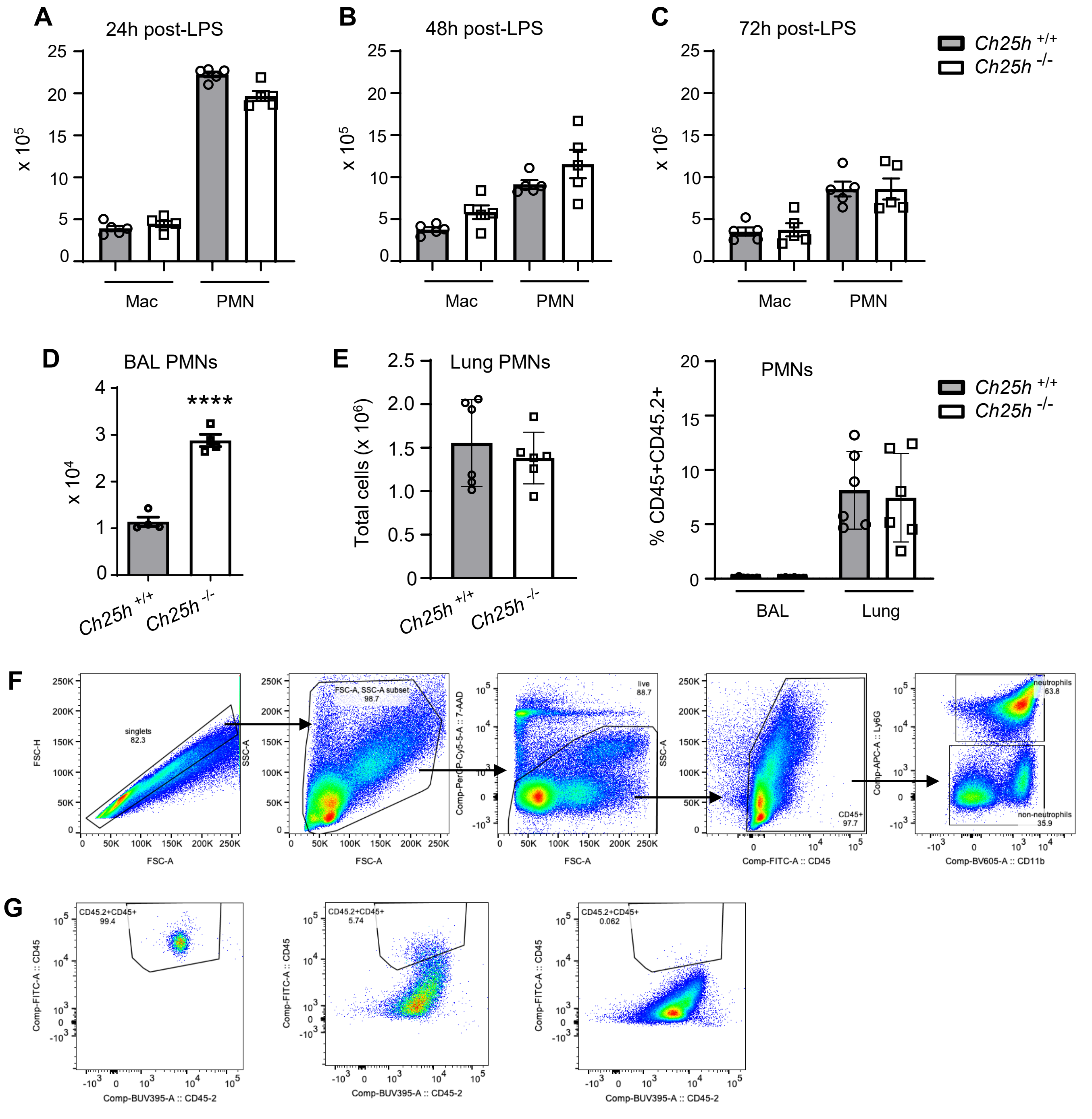

Figure S1. Temporal profile of BAL leukocyte differential after LPS inhalation. *Ch25h*$^{+/+}$ and *Ch25h*$^{-/-}$ mice were exposed to high-dose LPS aerosol. BAL was collected at 24h (**A**), 48h (**B**) and 72h (**C**) post-exposure and macrophages (Mac) and neutrophils (PMN) quantified as shown. (**D**) BAL PMNs were quantified 120h after high-dose LPS aerosol inhalation. N=4-5/genotype/timepoint. (**E-G**) At 48h post-high dose LPS inhalation, mice were injected i.v. with anti-CD45 antibody and sacrificed 10 min later. After perfusion, lungs were removed, digested, and stained with anti-CD45.2 and other FACS antibodies. Total lung PMNs were quantified (E, left). Among these, the frequency of intravascular (CD45+CD45.2+) PMNs was quantified (E, right [the balance of CD45-CD45.2+ PMNs are extravascular]). Exemplary gating for a BAL sample is shown in panel F, and in panel G, exemplary CD45.2 vs. CD45 gating within the PMN gate is shown for a peripheral blood sample (left), lung sample (middle), and BAL sample (right). Data represent mean +/- s.e.m. *, *P*<0.0001; by unpaired two-tailed t-test. Data derive from experiments independent of that shown in Fig. 1.

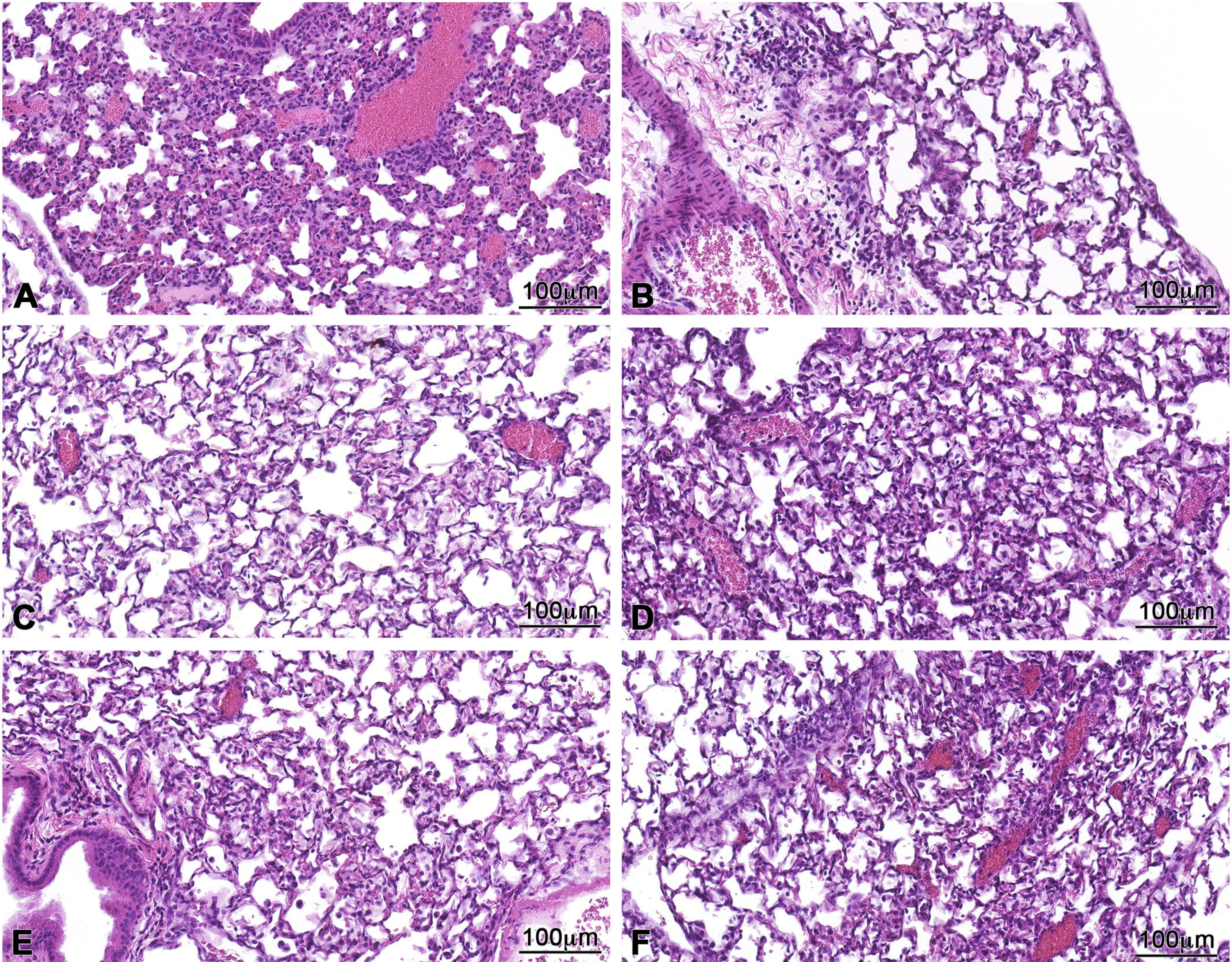
A
100μm
B
100μm
C
100μm
D
100μm
E
100μm
F
100μm

Figure S2. Wild type and Ch25h-null mice have equivalent lung histopathology after high-dose LPS inhalation challenge. Representative high-power images of H&E-stained lungs from *Ch25h*$^{+/+}$ (n=3) (**A-C**) and *Ch25h*$^{-/-}$ mice (n=3) (**D-F**) 48h post-inhalation of LPS.

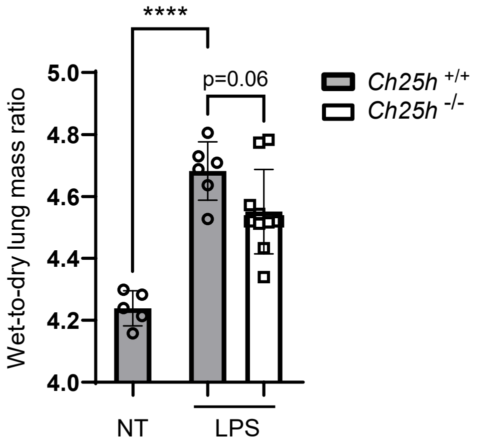

Figure S3. Pulmonary edema in Ch25h-null lungs as assessed by wet-to-dry ratio. *Ch25h*$^{+/+}$ and *Ch25h*$^{-/-}$ mice (n=5-9) were exposed to high-dose LPS inhalation. At 24h post-exposure, lungs were excised and weighed before ('wet') and after ('dry') vacuum desiccation, from which wet-to-dry mass ratio was calculated. Lungs were also analyzed from naïve (non-treated; NT) *Ch25h*$^{+/+}$ mice. Data are mean +/- s.e.m. and are representative of two independent experiments. ****, *P*<0.0001; by unpaired two-tailed t-test.

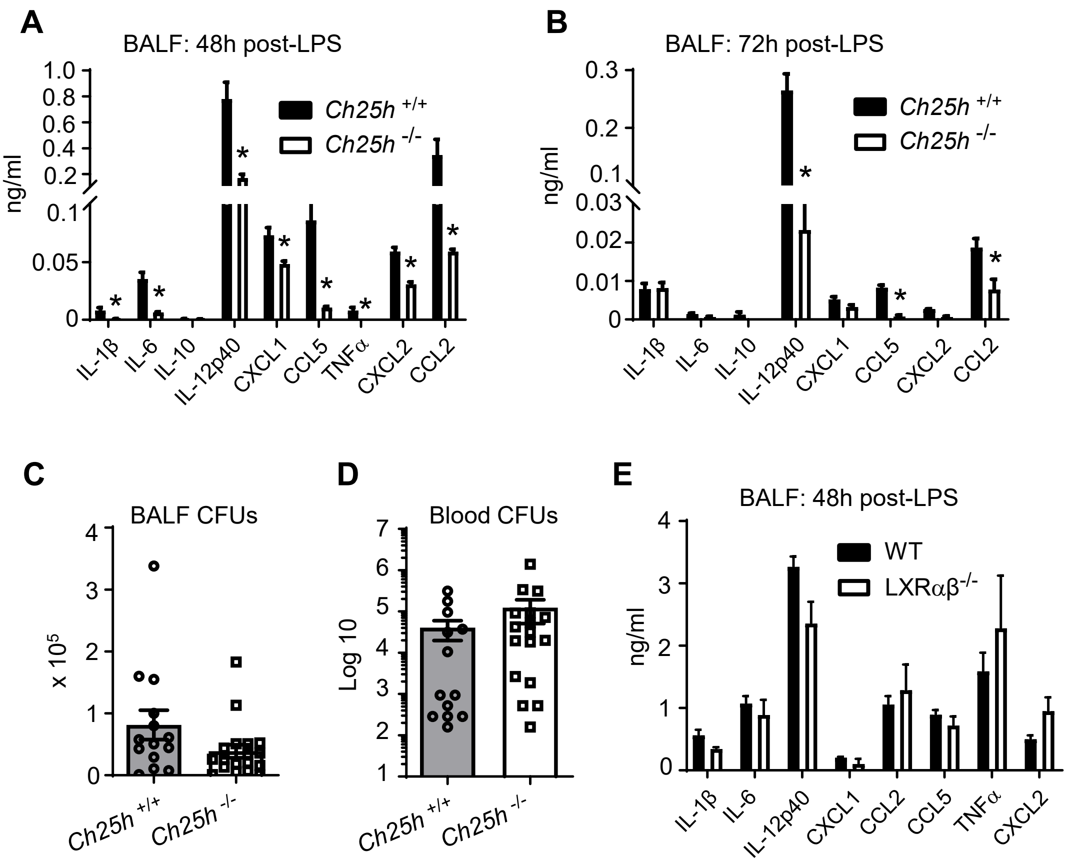

Figure S4. Reduced cytokine expression in *Ch25h-null* lungs after high-dose LPS challenge. (**A-B**) BALF from *Ch25h*$^{+/+}$ and *Ch25h*$^{-/-}$ mice (n=4/genotype/timepoint, repeated twice) was analyzed by multiplex assay for the indicated cytokines at 48h (A) and 72h (B) following inhalation of high-dose LPS. (**C-D**) Bacterial colony forming units (CFUs) were quantified in BAL (C) and peripheral blood (D) from mice 24h post-lung infection with *K. pneumoniae* (C: n=14-18/genotype; D: n=15-19/genotype; P=ns). (**E**) BALF cytokines were quantified in WT and LXR-null mice (n=5/genotype/timepoint, repeated twice) 48h after high-dose LPS inhalation. Data represent mean +/- s.e.m. *, P<0.05; by unpaired two-tailed t-test.

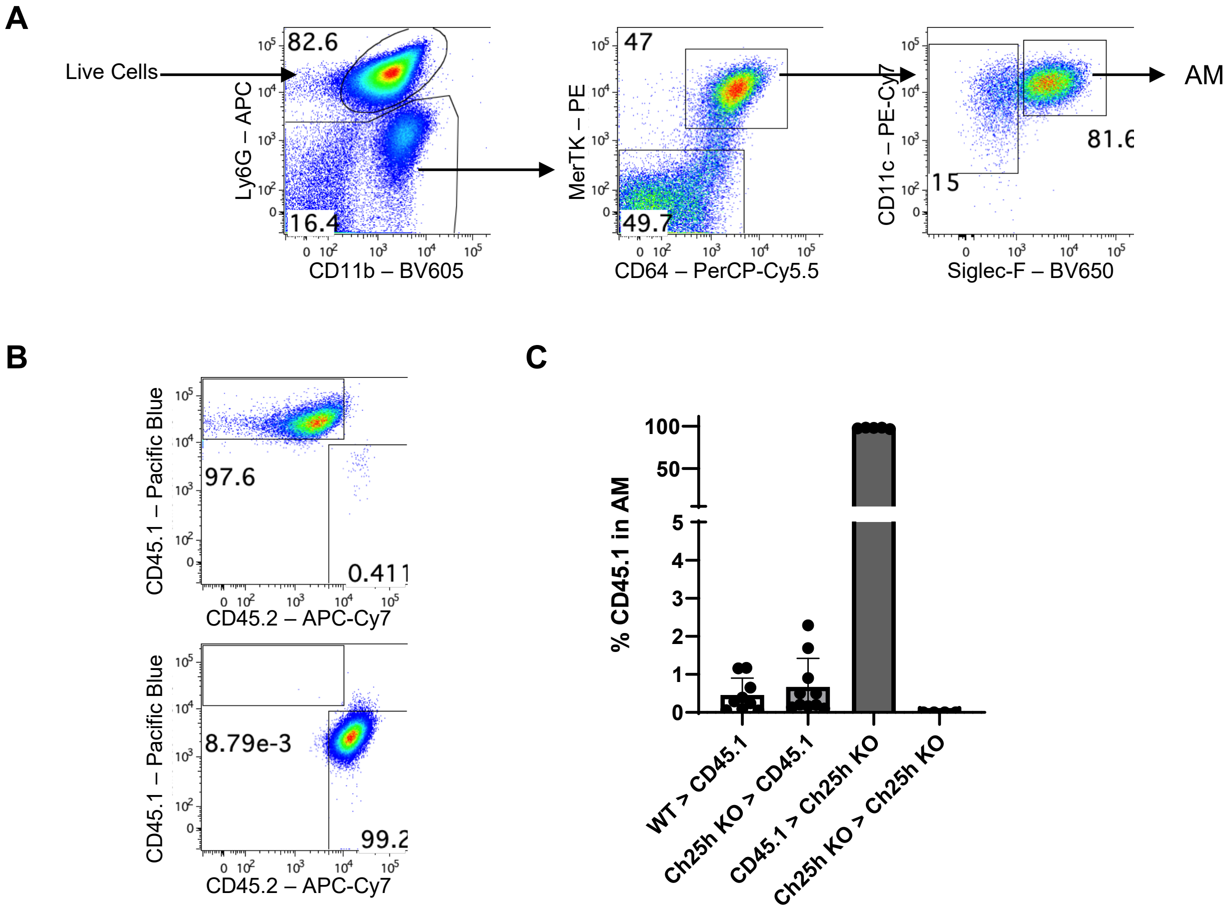

Figure S5. Confirmation of chimerism in bone marrow chimeric mice. Bone marrow chimeric mice for *Ch25h* were made through transfer of (CD45.1$^+$) *Ch25h*$^{+/+}$ or (CD45.2$^+$) *Ch25h*$^{-/-}$ bone marrow to irradiated (CD45.1$^+$) *Ch25h*$^{+/+}$ or (CD45.2$^+$) *Ch25h*$^{-/-}$ recipient mice. (**A**) BAL cells were collected from bone marrow chimeric mice 72h post-LPS inhalation (N=4-10/chimera). Exemplary FACS plots are shown for (A) resident AMs (MerTK$^+$CD64$^+$CD11c$^+$SiglecF$^+$Ly6G$^-$) and **(B)** CD45.1 vs. CD45.2 gating. (**C**) A summary plot of % CD45.1$^+$ cells in the AM gate of the four chimera groups, showing high (>97.5%) replacement of AMs with bone marrow-derived cells in all discernible cases (CD45.2$^+$ cell replacement in CD45.1 hosts [first two bars] and CD45.1$^+$ cell replacement in CD45.2$^+$ host [third bar]; Donor>Recipient; WT = C57BL/6).

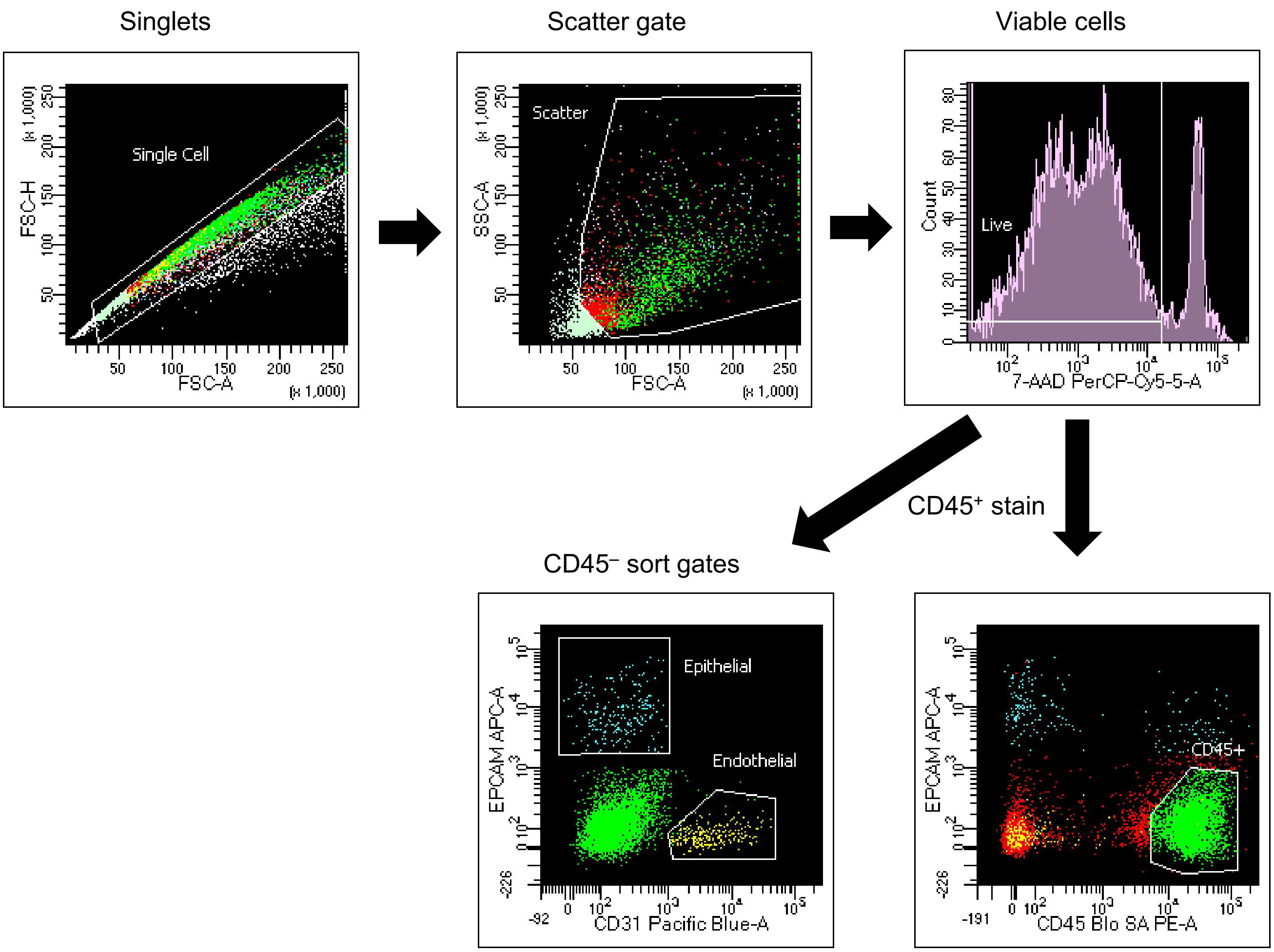

Figure S6. FACS gating strategy for isolation of pulmonary endothelial cells. As shown, endothelial cells were sorted from mouse lung digests as CD45⁻EpCAM⁻CD31⁺ cells.

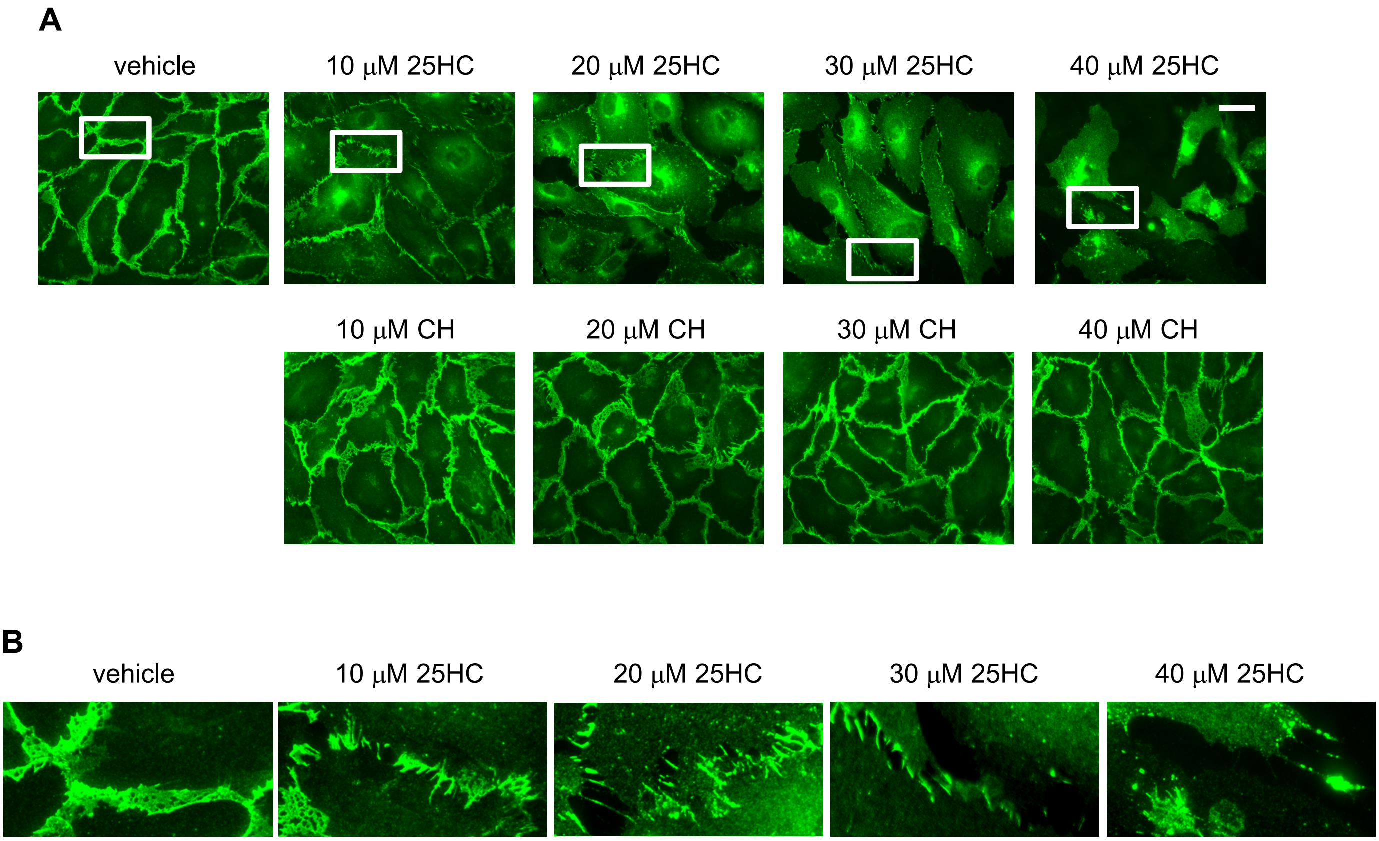

Figure S7. 25HC causes dose-dependent adherens junction disassembly. (A) Human pulmonary artery endothelial cells were treated with indicated concentrations of 25HC (top panels) or cholesterol (bottom panels) for 50h and immunofluorescence staining with VE-cadherin antibody was performed. **(B)** Inset boxes in panel A are displayed under higher magnification to show details of adherens junction disassembly. Images represent randomly selected 10-12 microscopic fields/condition from n=3 individual experiments. Bar: 10 μm.

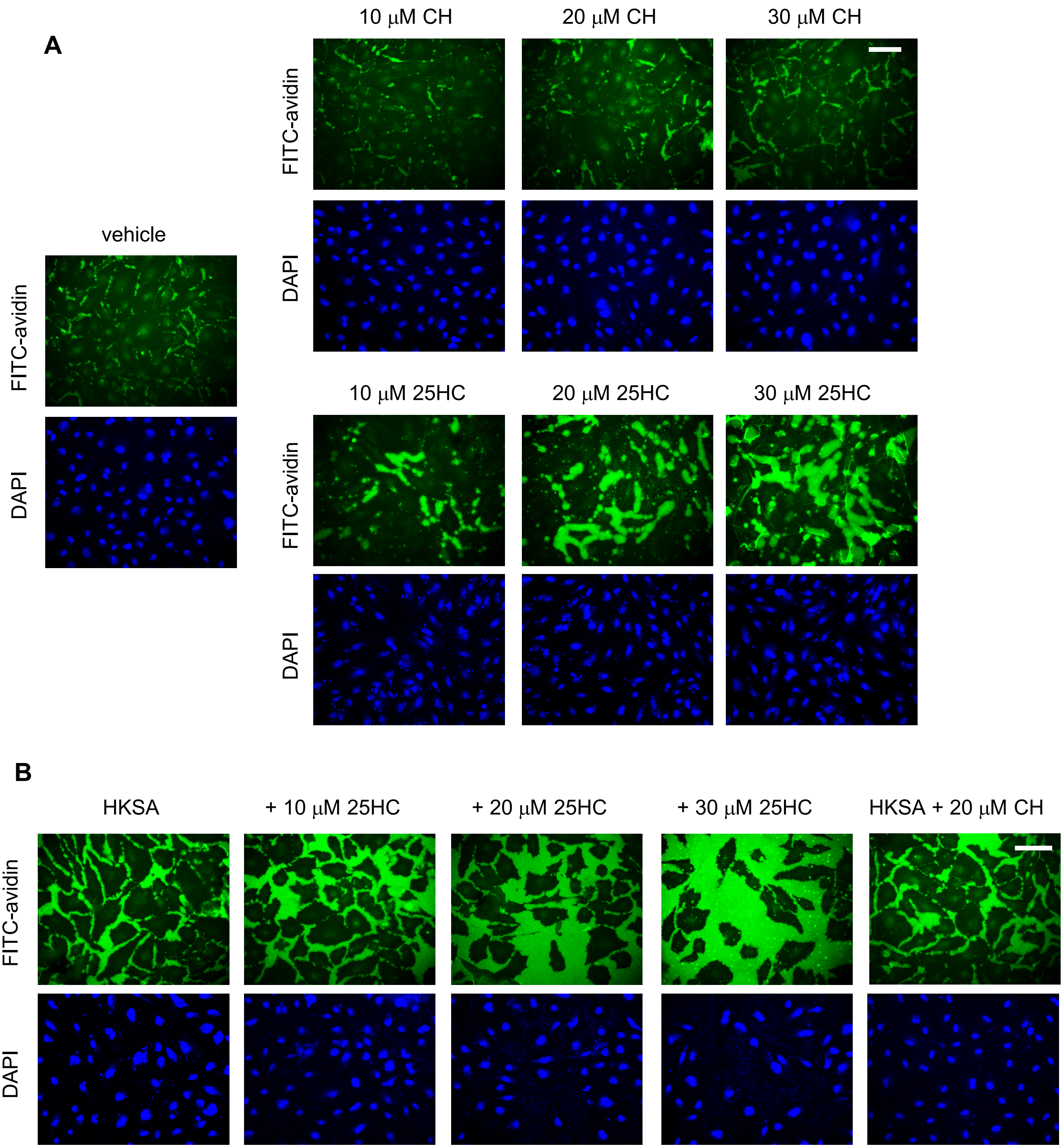

Figure S8. 25HC increases transendothelial permeability. (A) Human pulmonary artery endothelial cells were exposed to indicated concentrations of cholesterol (CH, top panel), 25HC (bottom panel), or vehicle (left) for 50h. (**B)** Human pulmonary artery endothelial cells were incubated with heat-killed S. aureus (HKSA; 2x10^8 particles/ml) for 30 min followed by treatment with indicated concentrations of 25HC or CH for 50h. In all cases, after treatment, permeability was assessed by addition of FITC-avidin to media, followed by imaging of FITC fluorescence bound to underlying biotin-coated matrix. DAPI counterstaining depicts cell nuclei. Images represent randomly selected 10-12 microscopic fields/condition from n=3 individual experiments. Scale bar = 20 μm.

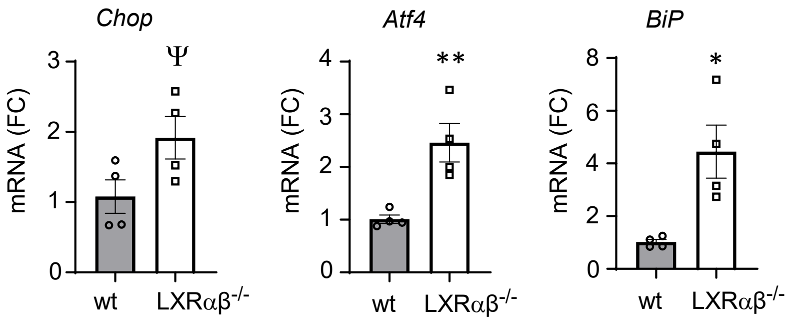

Figure S9. Upregulated markers of ER stress in lungs of LXR-null mice. Lung homogenates from wild type (wt) and LXR-null mice were analyzed by qPCR for the ER stress markers shown. N=4/genotype. Ψ, P=0.07; *, P<0.05; **, P<0.01 by unpaired two-tailed t-test. FC, fold change.

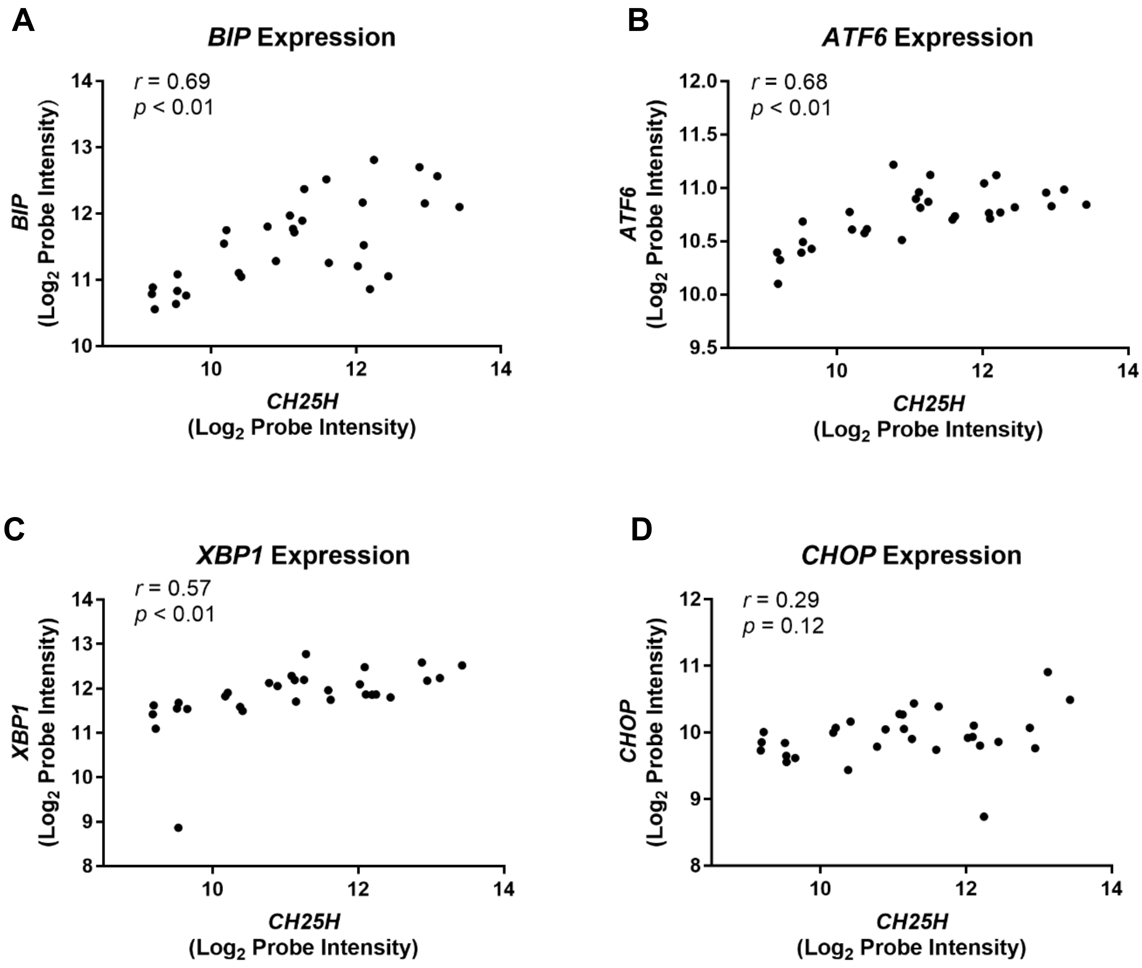

Figure S10. Alveolar macrophage *CH25H* expression correlates with ER stress genes in acute respiratory distress syndrome patients. Gene expression was quantified (microarray) in AMs harvested from ARDS patients (n=30) within 48h of ARDS diagnosis from a therapeutic trial of omega-3 fatty acids. A Pearson's test was used to generate a correlation coefficient between normalized $\log_2$ *CH25H* intensity and $\log_2$ *BIP* (A), *ATF6* (B), *XBP1* (C), or *CHOP* (D) probe intensities. The individual patient values are depicted.

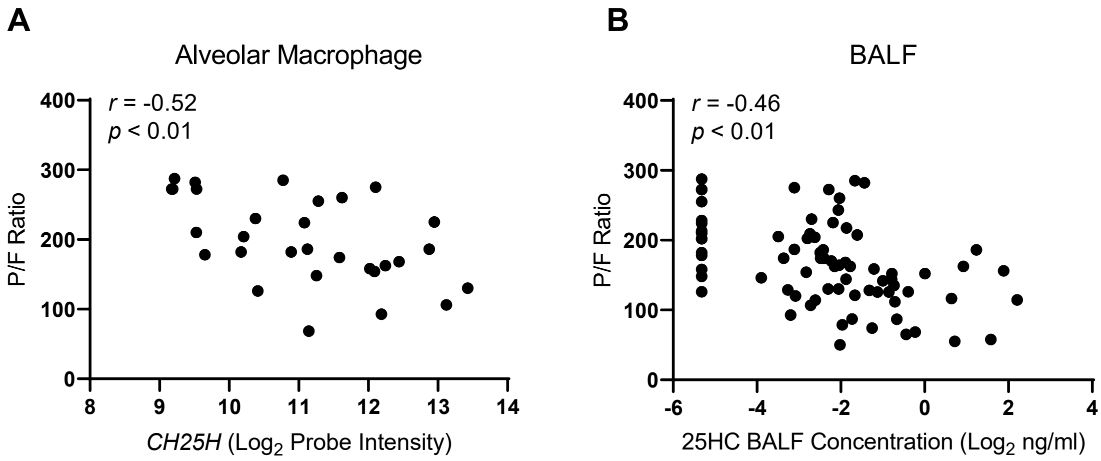

Figure S11. Correlation of AM CH25H and BALF 25HC to oxygenation in ARDS patients. Alveolar macrophage (AM) *CH25H* mRNA (quantified by microarray) (n=30) and bronchoalveolar lavage fluid (BALF) 25HC (quantified by mass spectrometry) (n=81) were measured in patients within 48h of ARDS diagnosis from a therapeutic trial of omega-3 fatty acids. A Pearson correlation coefficient (r) was calculated to assess the correlation between AM *CH25H* gene expression (A) or BALF 25HC levels (B) and PaO_2/F_IO_2 (P/F) ratio.

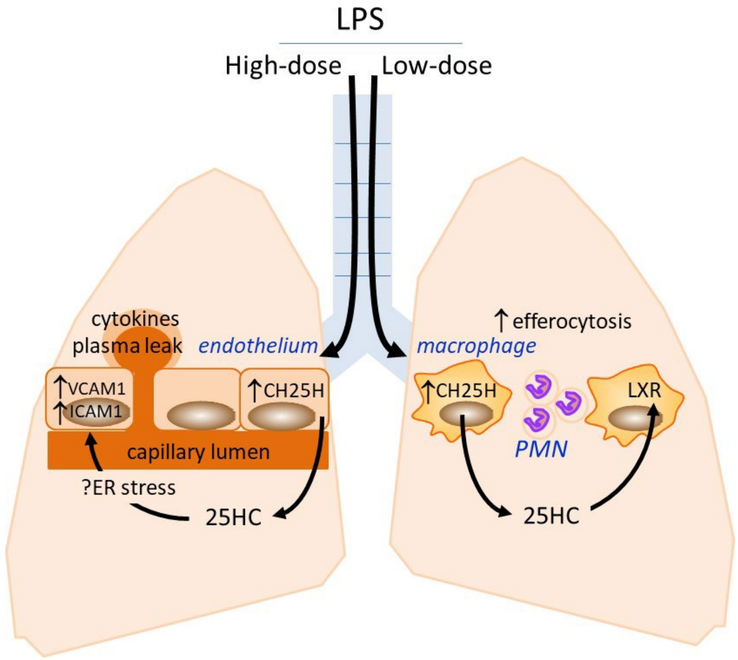

Figure S12. Proposed model for 25HC in lung inflammation and injury. In response to inhalation of low-dose LPS, alveolar macrophage CH25H induces 25HC, which activates Liver X Receptor (LXR)-dependent macrophage efferocytosis of apoptotic neutrophils (PMN), promoting resolution of airspace neutrophilia (Madenspacher et al., *JCI Insight* 2020 [PMID: 32343675]). As shown in the present report, high-dose LPS, a model of severe acute lung injury, induces endothelial upregulation of CH25H. Consequent locally generated 25HC activates endothelial cells, possibly through endoplasmic reticulum (ER) stress, inducing adhesion molecules and cytokines, and provoking adherens junction disassembly and paracellular plasma leak. CH25H = cholesterol-25-hydroxylase; 25HC = 25-hydroxycholesterol; ICAM1 = intercellular adhesion molecule-1; VCAM1 = vascular cell adhesion molecule-1.
